# A Segregating Structural Variant Defines Novel Venom Phenotypes in the Eastern Diamondback Rattlesnake

**DOI:** 10.1093/molbev/msaf058

**Published:** 2025-03-18

**Authors:** Pedro G Nachtigall, Gunnar S Nystrom, Emilie M Broussard, Kenneth P Wray, Inácio L M Junqueira-de-Azevedo, Christopher L Parkinson, Mark J Margres, Darin R Rokyta

**Affiliations:** Department of Biological Science, Florida State University, Tallahassee, FL, USA; Laboratório de Toxinologia Aplicada, CeTICS, Instituto Butantan, São Paulo, SP, Brazil; Department of Biological Science, Florida State University, Tallahassee, FL, USA; Department of Biological Science, Florida State University, Tallahassee, FL, USA; Biodiversity Center, University of Texas at Austin, Austin, TX, USA; Laboratório de Toxinologia Aplicada, CeTICS, Instituto Butantan, São Paulo, SP, Brazil; Department of Biological Sciences, Clemson University, Clemson, SC, USA; Department of Integrative Biology, University of South Florida, Tampa, FL, USA; Department of Biological Science, Florida State University, Tallahassee, FL, USA

**Keywords:** venom, rattlesnake, structural variation, conservation, genomics

## Abstract

Of all mutational mechanisms contributing to phenotypic variation, structural variants are both among the most capable of causing major effects as well as the most technically challenging to identify. Intraspecific variation in snake venoms is widely reported, and one of the most dramatic patterns described is the parallel evolution of streamlined neurotoxic rattlesnake venoms from hemorrhagic ancestors by means of deletion of snake venom metalloproteinase (SVMP) toxins and recruitment of neurotoxic dimeric phospholipase A2 (PLA2) toxins. While generating a haplotype-resolved, chromosome-level genome assembly for the eastern diamondback rattlesnake (*Crotalus adamanteus*), we discovered that our genome animal was heterozygous for a ∼225 Kb deletion containing six SVMP genes, paralleling one of the two steps involved in the origin of neurotoxic rattlesnake venoms. Range-wide population-genomic analysis revealed that, although this deletion is rare overall, it is the dominant homozygous genotype near the northwestern periphery of the species’ range, where this species is vulnerable to extirpation. Although major SVMP deletions have been described in at least five other rattlesnake species, *C. adamanteus* is unique in not additionally gaining neurotoxic PLA2s. Previous work established a superficially complementary north–south gradient in myotoxin (MYO) expression based on copy number variation with high expression in the north and low in the south, yet we found that the SVMP and MYO genotypes vary independently, giving rise to an array of diverse, novel venom phenotypes across the range. Structural variation, therefore, forms the basis for the major axes of geographic venom variation for *C. adamanteus*.

## Introduction

Structural variants (SVs) are a class of mutation affecting large (typically defined as >50 nucleotides) regions of a genome (Weischenfeldt et al. 2013) and include changes in chromosomal organization (e.g. translocations and inversions) or content (e.g. deletions and duplications). SVs can impact gene-expression patterns through duplication or deletion of genes, thereby increasing or decreasing mRNA and protein levels, and alter linkage patterns in affected regions (Mérot et al. 2020). SVs appear to be ubiquitous and are known to affect phenotypes critical to adaptation and speciation (Zhang et al. 2021b). For example, SVs are involved in high-altitude adaptation in humans (Shi et al. 2023), and a large chromosomal inversion was found to control tail length in deer mice, which is relevant for adaptation to forest or prairie habitats (Hager et al. 2022). Additionally, a 2.25 Kb retrotransposon insertion contributes to differences in plumage patterns involved in premating isolation in two subspecies of European crow (Weissensteiner et al. 2020). Despite such canonical examples, most SVs and their roles in evolutionary processes remain uncharacterized because technical challenges preclude their detection and study (Mérot et al. 2020; Xu et al. 2021). Read length and accuracy, in particular, limit the size and nature of SVs that can be detected with high confidence (Ho et al. 2020); many SVs are not detectable without long-read sequencing data (Chaisson et al. 2019).

Recent innovations in DNA sequencing and new bioinformatic approaches facilitate the generation and assembly of genomes at chromosome-level with haplotype resolution (Giani et al. 2020; Cheng et al. 2022). Specifically, the combination of PacBio HiFi sequencing, which results in reads >15 Kb with error rates of ∼1%, Hi-C, which produces short reads capturing 3-dimensional chromatin organization, and novel computational pipelines allows the generation of high-resolution genomes (Garg 2021; Rhie et al. 2021). Haplotype-resolved assemblies enable precise identification of all classes of genetic variation, from single-nucleotide polymorphisms (SNPs) to large SVs, including those in the heterozygous state in the source organism. The resolution of whole-genome haplotypes of species and populations has, in particular, revealed SVs affecting adaptive traits in diverse lineages (Chaisson et al. 2019; Low et al. 2020; Ebert et al. 2021; Hämälä et al. 2021; Garg 2023; Nakandala et al. 2023; Li et al. 2024a), suggesting that such approaches are critical for understanding phenotypic evolution.

Snake venoms are variable in composition at all phylogenetic scales (Casewell et al. 2020; Holding et al. 2021), yet the most phenotypically consequential known variants tend to involve large SVs segregating within species (Dowell et al. 2018). At the family level, major differences exist between the dominant types of toxins present. For example, elapids have venoms comprised largely of phospholipases A2 (PLA2s) and three-finger toxins (3FTxs), whereas the dominant components in viperid venoms tend to be snake venom metalloproteinases (SVMPs), snake venom serine proteases (SVSPs), and an independently recruited class of PLA2s (Oliveira et al. 2022). Among closely related species, SVs affecting numbers and identities of toxin-family paralogs commonly account for major venom differences (Dowell et al. 2016, 2018; Almeida et al. 2021; Margres et al. 2021; Nachtigall et al. 2022). Several rattlesnake species are polymorphic for vastly different venom phenotypes, with some populations expressing predominantly neurotoxic venoms and others expressing hemorrhagic venoms. Where examined, the underlying genetics involve distinct haplotypes for two venom-gene tandem arrays, PLA2s and SVMPs, that differ by major SVs (Dowell et al. 2018). The haplotypes are maintained such that the potent PLA2 haplotype, which encodes a dimeric PLA2 neurotoxin (Whittington et al. 2018), is nearly always associated with an SVMP haplotype with major portions of the SVMP array deleted. The maintenance of this polymorphism within species has been described in *Crotalus scutulatus*, *C. horridus*, and *C. helleri* (Dowell et al. 2018) and suggests that the loss of SVMP paralogs is beneficial only in the presence of a complementary neurotoxic PLA2 haplotype.

The eastern diamondback rattlesnake (*Crotalus adamanteus*) has one of the most thoroughly studied venoms of any animal (Rokyta et al. 2011, 2012; Margres et al. 2014, 2015a, 2015b; Rokyta et al. 2015; Wray et al. 2015; Margres et al. 2016a, 2016b, 2017a, 2017b; Rokyta et al. 2017; Margres et al. 2019; Schonour et al. 2020; Hogan et al. 2021; Harrison et al. 2022; Hogan et al. 2024), particularly at the genomic level, but still continues to yield novel insights into the mechanisms of venom evolution. *C. adamanteus* is the largest species of rattlesnake and is endemic to the southeastern United States, where it feeds primarily on mammals such as mice, rats, and rabbits (Means 2017). Its venom was among the first to be fully characterized by means of next-generation sequencing and proteomic approaches (Rokyta et al. 2011, 2012; Margres et al. 2014; Rokyta et al. 2015), and the species has been used to reveal patterns of geographic (Margres et al. 2015a, 2016a, 2016b, 2017b, 2019) and ontogenetic (Wray et al. 2015; Rokyta et al. 2017; Schonour et al. 2020) venom variation, the effects of hybridization on venom composition (Harrison et al. 2022), and the relationships between venom and other trophic adaptations (Margres et al. 2015b; Hogan et al. 2021). The major geographic pattern in venom variation previously described for this species involves a north–south gradient in copy number of the Myotoxin A (MYO) gene that correlates with a dramatic variation in the abundance of the encoded toxin in the venom, ranging from complete absence to a majority of the protein content (Margres et al. 2017a). Most recently, a chromosome-level genome assembly was described for *C. adamanteus* and used to characterize the gene-expression and epigenetic bases for its ontogenetic venom change (Hogan et al. 2024). Despite these substantial efforts, we are far from a comprehensive understanding of the patterns and causes of venom and genetic variation in this, or any, species.

The examination of patterns of genetic variation in *C. adamanteus* is of particular interest given growing concerns related to the conservation and management of this species, which is experiencing a rapid population decline and is considered vulnerable throughout much of its range (Waldron et al. 2013). *C. adamanteus* is state-endangered in North Carolina, a species of special concern in South Carolina, likely extirpated from Louisiana, and is currently under review for federal protection under the Endangered Species Act (Fish and Service 2012). This species is also included in the Department of Defense at-risk herpetofaunal species priority list. The rapid decline is primarily caused by anthropogenic forces (Means 2017), such as habitat loss and degradation, human persecution (Means 2009), and road mortality. Characterization of patterns of genetic variation, particularly fitness-related genetic variation (i.e. functional diversity, reviewed in Mable 2019), could, and perhaps should, provide the basis for targeted conservation and management efforts for this species.

We sequenced and assembled a chromosome-level, haplotype-resolved genome of *C. adamanteus* that fortuitously revealed a large SV spanning six SVMP genes between haplotypes. This SV substantially alters the venom proteomes of homozygotes for the deletion, suggesting a large phenotypic effect. Homozygotes for the deletion are rare range-wide but are the predominant genotype along the northwestern edge of the species’ range. The distribution of this deletion complements the previously described pattern in this species (Margres et al. 2017a) of copy-number variation for myotoxins to generate overlapping gradients of venom phenotypes across the species’ range, necessitating a reexamination of conservation strategies based on these patterns of fitness-related venom phenotypes.

## Results

### Chromosome-level Genome Assembly

**Fig. 1.**
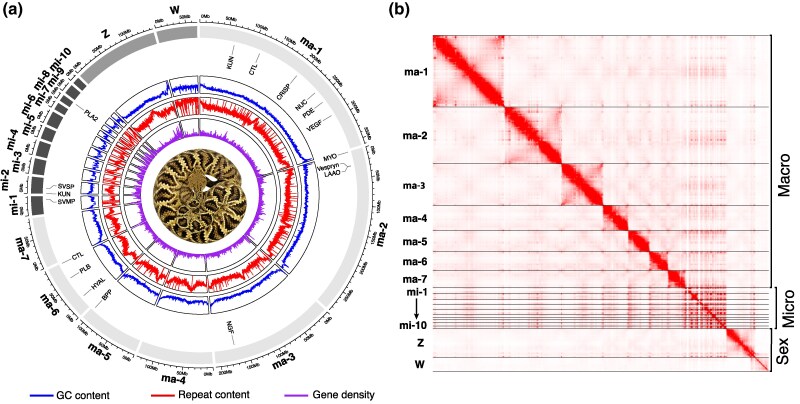
Overview of the genome assembly for *C. adamanteus*. a) A circos plot shows the distribution of venom genes across the genome. Circular rings from inner to outer display the gene density (purple), repeat content (red), and GC content (blue) within 100-Kb windows. The macro-, micro-, and sex chromosomes are colored in light gray, dark gray, and gray, respectively. b) An Hi-C contact map for all 19 assembled chromosomes, with darker colors indicating stronger interactions, indicates well-defined chromosomes consistent in structure and number with previous rattlesnake genome assemblies. Snake image credit: Michael P. Hogan.

Our *C. adamanteus* genome assembly, derived from a heterogametic female, comprised 19 chromosomes, including seven macrochromosomes (ma1–7), 10 microchromosomes (mi1–10), and both sex chromosomes (Z and W; Fig. 1a and b; Table 1), as expected for *Crotalus* species (Baker et al. 1972; Schield et al. 2019; Hogan et al. 2021; Margres et al. 2021; Hogan et al. 2024). We estimated a haploid genome size of 1.69 Gb and achieved a scaffold N50 of 208.8 Mb. The assembly was both highly accurate (QV score =44.7) and complete (BUSCO =95.7% complete, 94.6% single-copy, 1.1% duplicated, 0.9% fragmented, and 3.4% missing using the tetrapoda BUSCO gene set, which contains a total of 5,310 genes). Moreover, we identified telomeric repeats at both ends of 11 chromosomes and on one end of an additional five chromosomes, further attesting to the completeness of the assembly. Our primary assembly was similar to that previously published (Hogan et al. 2024) for *C. adamanteus* (Table 1), which was based on the same PacBio HiFi data but lower-coverage Hi-C data (∼5×) from a different individual. For our new assembly, we used a greater depth of Hi-C data (∼49×) from the same individual that was used for the HiFi data, allowing haplotype resolution. Our assembly statistics revealed that our genome assembly was of higher quality than any previously published snake genomes (Schield et al. 2019; Peng et al. 2020; Suryamohan et al. 2020; Li et al. 2021; Zhang et al. 2022; Peng et al. 2023).

**Table 1:** Statistics for the primary and haplotype assemblies of *C. adamanteus* compared with the previously published assembly (Hogan et al. 2024)

	Primary	Hap1	Hap2	Previous
Total size (Gb)	1.69	1.52	1.62	1.69
Scaffold N50 (Mb)	208.8	207.8	208.2	208.9
Contig N50 (Mb)	57.8	45.3	29.4	67.5
No. chromosomes	19	18	18	19
Sex chromosome	ZW	W	Z	ZW
GC content (%)	39.9	39.9	39.9	39.9
BUSCO (%)^a^	95.7	89.9	95.7	95.7
Quality value (QV) score	44.4	40.4	43.4	42.8
Genes^b^	34,471	32,461	34,255	21,841
	(20,156)	(17,861)	(19,470)	(17,810)
Venom genes	77	63	73	134

^a^Percentage of complete BUSCO genes using the tetrapoda gene set (odb10; total of 5,310 genes).

^b^Distinct gene-annotation approaches were applied in these studies. The number of genes matching the ENSEMBL database are provided in parentheses.

Repeats accounted for 52.61% of the genome, including 7.09% tandem repeats and 44.17% transposable elements (TEs). Among the TEs, 22.11%, 11.98%, and 7.39% were LINE, LTR, and DNA (i.e. DNA and MITE) TE families, respectively. To check for evidence of recent TE activity, we estimated the repeat landscape by calculating Kimura substitution levels (supplementary fig. S1, Supplementary Material online). We found evidence for a recent burst of TE activity from the LINE family, suggesting that these TEs are relevant for the genome dynamics and evolution of *C. adamanteus*. The high abundance and recent bursts of LINE families are in accordance with a previous comparative analysis of repeat sequences in squamates (Pasquesi et al. 2018), which showed that specific LINE elements may be responsible for rearrangement events in snake genomes.

We used funannotate (Palmer and Stajich 2017) to annotate 34,471 protein-coding genes, of which 15,037 were attributed to meaningful functional annotation beyond “hypothetical protein.” A similarity search revealed that 20,156 of the 34,471 (58.47%) predicted protein-coding genes matched with high identity and coverage to entries in the ENSEMBL database. Using ToxCodAn-Genome (Nachtigall et al. 2024), we identified 77 venom protein-coding genes (supplementary table S1, Supplementary Material online). These numbers differ from the previous assembly (Hogan et al. 2024, supplementary tables S2 and S3, Supplementary Material online), because we only included genes with medium to high expression in the venom-gland transcriptomes relative to transcriptomes from other tissues and that have been confirmed proteomically in vipers (Oliveira et al. 2022). The venom-gland transcriptome from the genome individual showed that the major components of the venom were MYOs, SVSPs, PLA2s, C-type lectins (CTLs), and SVMPs (supplementary fig. S2, Supplementary Material online), consistent with previous studies (Rokyta et al. 2012; Margres et al. 2014, 2015a, 2016a; Rokyta et al. 2017). The SVMPs, SVSPs, and PLA2s were each organized in single tandem arrays and located on microchromosomes, whereas MYO and CTLs were each organized in single tandem arrays and located on macrochromosomes, as previously reported in other *Crotalus* genomes (Schield et al. 2019; Hogan et al. 2021, 2024; Margres et al. 2021).

### Rearrangements Primarily Affected Intergenic Regions

**Fig. 2.**
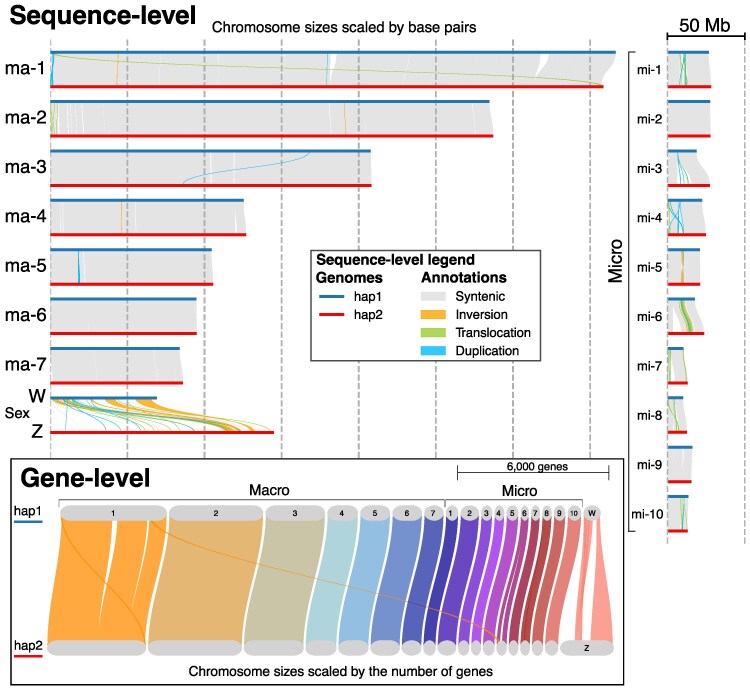
Sequence-level and gene-level synteny analyses between haplotypes of the *C. adamanteus* genome. For the sequence-level analysis, the chromosome sizes are scaled in base pairs, where the distances between the vertical gray dashed lines are 50 Mb. For the gene-level analysis, the chromosome sizes are scaled by the number of genes. Both sequence-level and gene-level analyses reveal that most rearrangements are occurring in intergenic regions, which may be a result of recent bursts of TE activity. The large numbers of rearrangements in the sex chromosomes are in agreement with previous cytogenetic studies in snakes (Matsubara et al. 2006; Viana et al. 2019).

The two haplotype assemblies returned different statistics relative to the primary assembly due to distinct rearrangements and associated sex chromosomes (Table 1). Their QV scores (>40) and Hi-C interaction maps indicated robust assemblies for both haplotypes (supplementary fig. S3, Supplementary Material online). Haplotype 1 (hap1) included the W chromosome, and haplotype 2 (hap2) included the Z chromosome. A synteny analysis revealed that the haplotypes were predominantly syntenic, with specific genomic regions showing rearrangements (Fig. 2). Rearrangements were primarily detected at the sequence-level (i.e. alignment of sequences between haplotypes, independent of gene presence/absence), and almost no rearrangements were observed affecting genes (i.e. when comparing gene positions between haplotypes) for macro- and microchromosomes. Together, these results indicate that most rearrangements were detected in intergenic regions, and these were enriched in microchromosomes and sex chromosomes (supplementary fig. S4A, Supplementary Material online). For all chromosomes, the regions containing rearrangements also showed a higher content of repetitive elements (supplementary fig. S4B, Supplementary Material online), suggesting that TE elements may play a role in generating these rearrangements.

The Z and W sex chromosomes presented high proportions of rearrangements at the sequence-level, which resulted in some rearrangements in gene locations along the chromosomes. Such rearrangements were previously reported by cytogenetic studies in snakes (Matsubara et al. 2006). The W chromosome showed the highest proportion of repeat-element-derived sequences (∼85%; supplementary fig. S5, Supplementary Material online), far exceeding the corresponding value for the Z chromosome (∼55%). Other snakes (Viana et al. 2019; Schield et al. 2022) show similar enrichment of repeat elements on the W chromosome. These results suggest that the W chromosome exhibits dynamic evolution and that the Z chromosome is more stable. However, further studies assembling telomere-to-telomere sex chromosomes of snake species may help confirm such features and reveal the evolutionary history of the sex-determination system in caenophidian snakes.

### Venom-gene Haplotypes Revealed a Large SV in the SVMP Array

To determine whether venom genes were affected by SVs in the heterozygous state for our genome animal, we compared these regions in the two haplotype assemblies (hap1 and hap2), and in the primary assembly (Table 2; supplementary table S1, Supplementary Material online). We detected no differences for single-paralog toxin genes between haplotypes (Table 2). Among the multiparalog toxin-gene arrays, the PLA2, CTL, and MYO arrays were consistent across haplotypes (Table 2; supplementary fig. S6, Supplementary Material online), whereas the SVSP and SVMP arrays showed apparent gene-content differences. To assess whether the latter two putative SVs were real or assembly artifacts, we evaluated read alignments and examined relationships among paralogs.

**Table 2:** Numbers of paralogs within each toxin gene family in primary and haplotype genome assemblies of *C. adamanteus*

	Primary	Haplotype 1	Haplotype 2
BPP	1	1	1
CRISP	1	1	1
CTL	10	10	10
HYAL	1	1	1
KUN	2	2	2
LAAO	2	2	2
MYO	4	4	4
NGF	1	1	1
NUC	1	1	1
PDE	1	1	1
PLA2	3	3	3
PLB	1	1	1
SVMP	23	17	23
SVSP	24	16	20
VEGF	1	1	1

Abbreviations: BPP, bradykinin-potentiating peptide; CRISP, cysteine-rich secretory protein; CTL, C-type lectin; HYAL, hyaluronidase; KUN, Kunitz-type protease inhibitor; LAAO, L-amino acid oxidase; MYO, myotoxin/crotamine; NGF, nerve growth factor; NUC, nucleotidase; PDE, phosphodiesterase; PLA2, phospholipase A_2_; PLB, phospholipase B; SVMP, snake venom metalloproteinase; SVSP, snake venom serine protease.

The differences in paralog content between assemblies in the SVSP gene array (Table 2) were likely due to assembly artifacts. The primary assembly had 23 SVSP paralogs, whereas hap1 and hap2 had 14 and 19 paralogs, respectively. Further inspection revealed that this region contained signal for being error-prone according to VerityMap and showed collapsed reads in the breakpoints defining the differences (supplementary figs. S10 and S11, Supplementary Material online). Moreover, the paralog phylogeny showed that variable SVSP paralogs were identical to paralogs shared by all assemblies, indicating either recent duplications or assembly artifacts (supplementary fig. S12, Supplementary Material online). Because we could not reject assembly artifacts as the basis for the assembly differences in this region, we did not pursue this potential SV further.

**Fig. 3.**
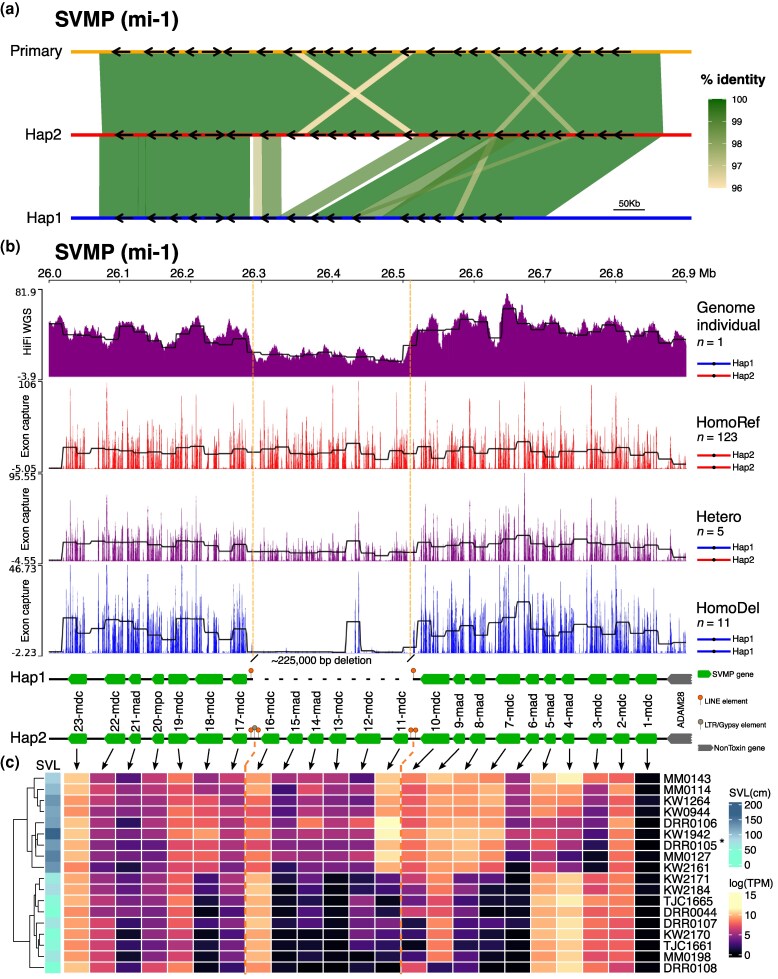
Genomic alignment, genomic read coverages, and expression levels of SVMPs. a) Riparian plot showing the genomic alignment of SVMP loci between the primary assembly (orange) and each haplotype (red and blue). Black arrows represent each SVMP gene in that specific assembly. The shaded areas represent the percentage identity of alignments obtained through BLAST; alignments were filtered to sizes >10 Kb and percentage identity >95%. b) SVMP coverage in the genome individual (heterozygote), using the HiFi whole-genome data, and the other 139 individuals, using exon-capture sequencing data. Example coverage tracks of five individuals each of HomoRef (homozygotes for the entire SVMP array), Heterozygotes, and HomoDel (homozygotes for the SVMP deletion) are displayed in red, purple, and blue, respectively. Black lines represent average coverage across all individuals in that class in 20-Kb sliding windows. The vertical, dashed orange lines indicate the SVMP deletion region. At the bottom, the alignment of both haplotypes shows the ∼225 Kb deletion in hap1 and the transposable elements (TE) flanking the deleted region. Only TEs at the deletion boundaries are shown. c) SVMP expression levels in the venom-gland transcriptomes of nine adults (snout-to-vent length >100 cm) and nine juveniles (snout-to-vent length <100 cm) show that the deleted paralogs include ontogenetically regulated genes. The genome individual (DRR0105) is indicated with an asterisk. Abbreviations: SVL, snout-to-vent length; TPM, transcripts per million.

We identified a large deletion in the SVMP array in hap1 relative to hap2 and the primary assembly (Fig. 3a), which comprised ∼225 Kb and six SVMP paralogs (*SVMP-11-mdc*, *SVMP-12-mdc*, *SVMP-13-mdc*, *SVMP-14-mad*, *SVMP-15-mad*, and *SVMP-16-mdc*). VerityMap confirmed that the SVMP loci in all assemblies did not occur in error-prone regions (supplementary fig. S7, Supplementary Material online). Read coverage for all assemblies corroborated the VerityMap output by presenting a similar pattern along the SVMP region in the primary assembly and both haplotype assemblies. We found no evidence for collapsed regions in the SVMP array for all assemblies (supplementary fig. S8, Supplementary Material online). Moreover, the SVMP paralog phylogeny revealed that none of the SVMP paralogs were identical copies (supplementary fig. S9, Supplementary Material online), providing additional evidence in support of the heterozygous SV in this region. Our data therefore provided a robust characterization of a six-paralog deletion affecting the SVMP toxin array for one of the haplotypes of our genome individual.

We identified conserved LINE elements at both boundaries of the SVMP deletion, as well as one LTR/Gypsy element surrounded by these LINE elements at one end (Fig. 3b). The recent genome-wide burst of LINE and LTR element activity in *C. adamanteus* (supplementary fig. S1, Supplementary Material online) suggests an active role for these elements in shaping the genome of this species in addition to a specific role in the evolution of the SVMP array. TEs have been hypothesized to be responsible for rearrangements, such as duplications, deletions, and gene fusions of SVMP genes in other *Crotalus* species (Giorgianni et al. 2020).

### The SVMP Deletion is Rare but Regionally Abundant

**Fig. 4.**
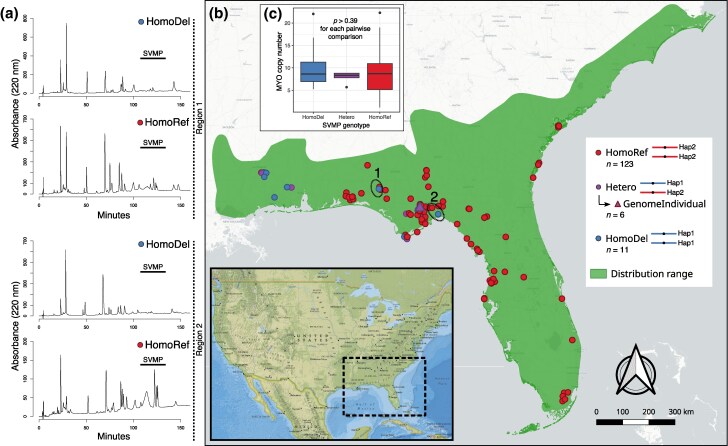
The geographic distribution of the SVMP deletion and the phenotypic differences between homozygotes of each genotype. a) Reversed-phase high-performance liquid chromatography (RP-HPLC) of individual venoms from animals confirmed to be homozygous for each SVMP genotype from two different regions demonstrate clear differences in SVMP content. Region 1 was Eglin Air Force Base, and region 2 was the Apalachicola National Forest. b) Sampling distribution of specimens relative to the overall species range. Red dots represent individuals that were confirmed as homozygous for the wild-type SVMP region (HomoRef) on the basis of exon-capture data. Purple circles represent heterozygous (Hetero) individuals. Blue circles represent individuals that were homozygous for the SVMP deletion (HomoDel). The purple triangle represents the genome individual, which was a heterozygote for the deletion. c) Estimated copy number of MYO genes in individuals genotyped for the SVMP deletion. We found no statistically significant differences between groups using the Wilcoxon rank sum test.

To assess the geographic distribution of the SVMP deletion, we used anchored hybrid enrichment sequencing data designed to capture the exons of toxin and nontoxin genes (Margres et al. 2019) from 139 *C. adamanteus* individuals from across the species’ range (Fig. 3b and Fig. 4). On the basis of mapping coverage, we were able to categorize each individual as homozygous for the full SVMP array (HomoRef), heterozygous (Het), or homozygous for the deletion (HomoDel). We detected 123 HomoRef, 5 Het, and 11 HomoDel individuals, indicating that the SVMP deletion haplotype was rare across the entire range. HomoDel and Het individuals were primarily restricted to the northwestern edge of the species’ distribution and were the dominant genotypes in Mississippi (Fig. 4b).

We observed anomalously high coverage in the last exons of the *SVMP-12-mdc* gene in HomoDel individuals despite this gene being within the deletion (Fig. 3b). These peaks resulted from multimapped reads due to a high similarity between *SVMP-12-mdc* and nondeleted paralogs *SVMP-17-mdc* and *SVMP-19-mdc* (supplementary fig. S9A, Supplementary Material online). *SVMP-12-mdc* shows 92.9% overall identity with *SVMP-17-mdc* and 91.5% identity with *SVMP-19-mdc*. In particular, the region of *SVMP-12-mdc* with reads mapping (the last five exons) shows the highest identity with *SVMP-17-mdc* (97.5%; supplementary fig. S9B, Supplementary Material online). Higher-than-average-coverage peaks in this region of the *SVMP-12-mdc* gene are also present in Het and HomoRef individuals. The peaks observed in last exons of *SVMP-12-mdc* of HomoDel individuals comprised ∼95% multimapped reads, whereas the average of multimapped reads for this gene in HomoRef and Het individuals was ∼80%. These anomalous peaks therefore represent read-mapping artifacts due to recently derived SVMP paralogs and conserved exons.

### The SVMP Deletion Affects the Venom Phenotype

To assess the potential for phenotypic effects of the deletion, we analyzed the venom-gland transcriptomes of 18 individuals (Fig. 3c) to estimate typical expression levels for the deleted genes in wild-type individuals. On the basis of their sampling localities (supplementary table S4, Supplementary Material online) and their high expression of the deleted paralogs, these 18 individuals should all be HomoRef or Het genotypes. Two SVMP paralogs within the deletion (*SVMP-11-mdc* and *SVMP-12-mdc*) were highly expressed in adults (snout-to-vent length >100 cm; Waldron et al. 2013), and one paralog (*SVMP-16-mdc*) was highly expressed in juveniles (snout-to-vent length <100 cm; Waldron et al. 2013). Previous work showed that *SVMP-11-mdc* and *SVMP-12-mdc* were up-regulated in adults and that *SVMP-16-mdc* was up-regulated in juveniles (Hogan et al. 2024). Not only are some of the SVMP paralogs highly expressed, but some are involved in the fine-tuning of venom composition across life history. Individuals heterozygous or homozygous for the deletion therefore potentially show unique venom phenotypes for both adults and juveniles.

**Fig. 5.**
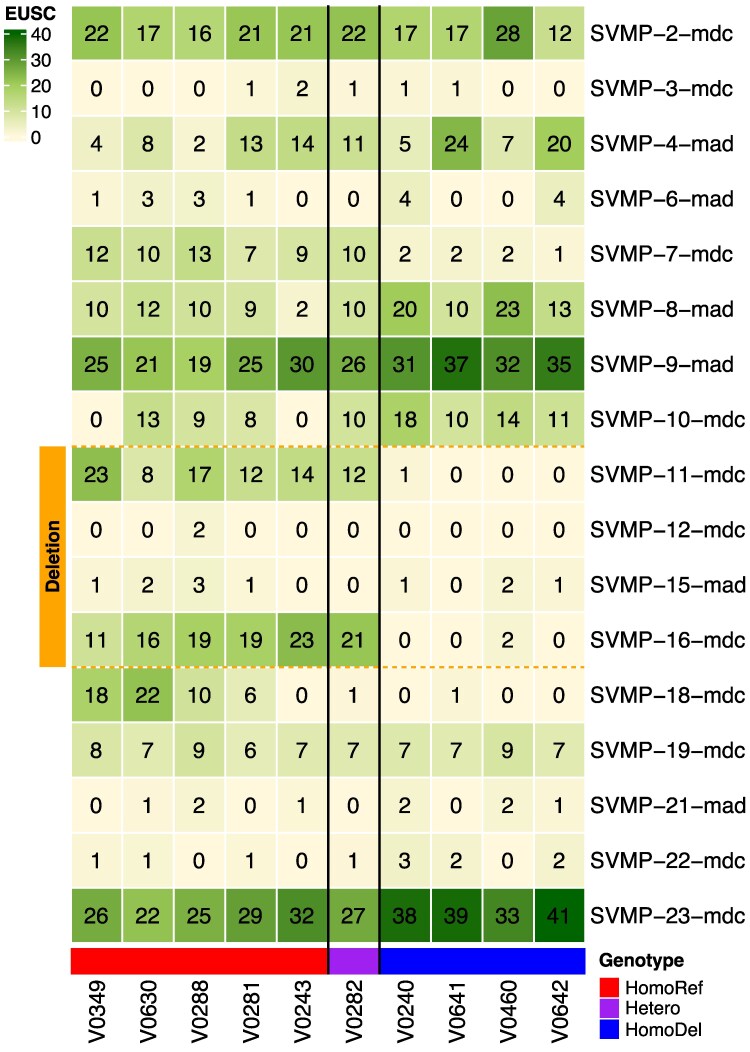
SVMP proteomic abundances from venoms of genotyped individuals. The heatmap shows the normalized Exclusive Unique Spectra Count (EUSC) for SVMPs with at least one unique peptide matching in at least one sample. Deleted SVMPs are represented with orange colored squares on the left. The genotype of each sample is at the bottom, where red indicates homozygotes for the entire wild-type SVMP array (HomoRef), purple indicates heteorozygotes (Hetero), and blue indicates homozygotes for the deletion (HomoDel). At least two of the six deleted SVMPs have major effects on venom proteomic composition.

To directly measure the effects of the SVMP deletion on venom composition, we performed reverse-phase high-performance liquid chromatography (RP-HPLC) and mass spectrometry (MS) on venoms from genotyped individuals. We first visually compared representative homozygotes of each genotype from the same populations by means of RP-HPLC (Fig. 4a) to show that HomoRef and HomoDel individuals have striking differences in the SVMP peak region (i.e. peaks eluting at ∼120 min; Margres et al. 2014). We then performed MS analyses on adult venoms from five HomoRef, one Het, and four HomoDel individuals. We detected unique proteomic signal for 17 SVMP paralogs, including four paralogs within the deletion (Fig. 5). For many of these paralogs, however, the signal was at a low background level (i.e. ≤2 Exclusive Unique Spectra Counts), leaving their presence in the venom unconfirmed. The two most highly expressed deletion paralogs identified in the transcriptomic data above (*SVMP-11-mdc* and *SVMP-16-mdc*) were, as expected, abundant in the venoms of HomoRef and Het individuals, but not HomoDel individuals; we found no proteomic evidence for any of the four detected deletion paralogs in the venoms from HomoDel individuals (supplementary fig. S13, Supplementary Material online).

### Independence between the SVMP Deletion and MYO Copy Number

Previous work in *C. adamanteus* (Margres et al. 2017a) described substantial variation in copy number for the MYO gene with corresponding dramatic effects on venom composition. The general trend was that MYO was at low copy number or absent from the genomes of individuals in the southern portion of the range and at high copy number in the north of the range, superficially complementing our pattern for the SVMP gene array (Fig. 4). To test for a correlation between SVMP and MYO genotypes, we estimated MYO copy number from the hybrid-enrichment data for the same individuals genotyped for the SVMP deletion. We found no statistically significant relationship between MYO copy number and SVMP genotype (Fig. 4c and supplementary S14, Supplementary Material online). The comparison between homozygotes returned a *P*-value of 0.39, whereas comparisons between the heterozygotes and homozygotes for the presence and deletion of the SVMP paralogs resulted in *P*-values of 0.70 and 0.59, respectively. The SVMP deletion distribution showed a clear increase in frequency toward the western periphery of the range, with no detected occurrences east of the Suwannee River (supplementary fig. S14, Supplementary Material online), and we confirmed the complementary pattern of MYO copy-number increasing from south to north (supplementary fig. S14, Supplementary Material online). However, rather than finding evidence for linkage disequilibrium between the regions, we find apparent independence, suggesting that the prevalence of the SVMP deletion in the northwestern region of the range (Mississippi) is not contingent on the presence of high-copy-number MYO haplotypes. Furthermore, this lack of association indicates that a large component of geographic variation in the venom phenotype of *C. adamanteus* was generated by two independent gradients in the frequencies of SV-based haplotypes on two different chromosomes.

## Discussion

Biases in mutational processes can be as determinative of evolutionary outcomes as the selective pressure acting on the resulting mutations (Rokyta et al. 2005; Sackman et al. 2017; Stoltzfus and McCandlish 2017; Cano et al. 2023). Observations of the genetic bases of beneficial phenotypes can provide critical information about whether certain types of mutations are more likely to contribute to adaptation by virtue of some combination of their rates of occurrence and phenotypic consequences. Because the genes encoding venom components are readily identifiable, we know that positive selection is rampant within these coding sequences (Lynch 2007; Gibbs and Rossiter 2008; Rokyta et al. 2011). Expression differences, however, are also widespread in venoms and may be the quickest characteristics to evolve (Margres et al. 2016a, 2017a), possibly because they can be accomplished by means of multiple mutational mechanisms, including gene duplication and deletion and changes to multiple *cis*-regulatory regions. Only as high-contiguity genome assemblies become available for venomous species are we able to begin to comprehensively assess the full spectrum of mutational types, and SVs are emerging as a major component of intraspecific variation for many species, including *C. adamanteus*. Structural variants represent a large proportion of genetic variants affecting phenotypes in eukaryotes (Ho et al. 2020), but their relative contributions to trait evolution are largely unknown. Starting with a single genome assembly of sufficient quality to identify large SVs in the heterozygous state, we uncovered a new SV with a major impact on venom variation for *C. adamanteus*. Although the majority of SVs are likely to be deleterious due to their impacts on gene expression and recombination rates, they are also known to contribute to adaptation (Tigano et al. 2018; Vickrey et al. 2018; Catanach et al. 2019; Faria et al. 2019; Todesco et al. 2020; Weissensteiner et al. 2020; Hämälä et al. 2021; Hager et al. 2022; Saitou et al. 2022; Shi et al. 2023; Li et al. 2024b). We showed that a large SV affecting six toxin genes is locally dominant in a population of *C. adamanteus*, which, in conjunction with parallel occurrences of similar deletions in other rattlesnake species, suggests it confers a local fitness advantage. We also demonstrated that multimapped short reads can potentially confound coverage-based genotyping for SVs involving genes within tandem duplicate arrays (like those containing many, if not most, venom genes). Shared, conserved exons among paralogs can result in spurious signal for the presence of deleted paralogs. For our data, the spurious signal was limited to a small minority of exons in a single-deleted paralog and was therefore straightforward to exclude as an alignment artifact.

Parallel evolution (Ventura et al. 2011; Pearse et al. 2014; Bohutínská et al. 2021) occurs when similar phenotypes evolve independently in two distinct lineages in response to similar selective pressures. The large SVMP deletion we identified in *C. adamanteus* parallels similar deletions in this same genomic region in multiple other *Crotalus* species (supplementary figs. S15 and S16, Supplementary Material online; Giorgianni et al. 2020; Margres et al. 2021). The SVMP deletion of *C. adamanteus*, however, was not correlated with the acquisition of neurotoxic PLA2s as in every other reported case (Dowell et al. 2016, 2018; Margres et al. 2021). A dichotomy in venom types for rattlesnakes has long been hypothesized, with type I venoms showing high metalloproteinase activity and low neurotoxic activity and type II venoms showing the inverse (Mackessy 2010). Individuals of *C. adamanteus* with the SVMP deletion appear to depart from this pattern (Fig. 4a).

MYO expression also shows parallel patterns of extreme expression variation within other *Crotalus* species, including *C. scutulatus* (Strickland et al. 2018) and *C. viridis* (Smith et al. 2023). Remarkably, for *C. viridis*, MYO expression is almost perfectly inversely correlated with SVMP expression levels (Smith et al. 2023), although the genetic basis for this phenotypic variation is not known. This pattern suggests that MYO activity could replace neurotoxic PLA2 activity in some scenarios (Smith et al. 2023). In contrast to the results from *C. viridis*, however, we found no statistical association between the genotypes underlying high MYO expression and low SVMP expression. This suggests a more complex pattern of geographic variation in selective pressures as well as a remarkable mosaic of SV-based phenotypes in these venoms that does not neatly partition into a distinct dichotomy.

The geographic regions with the highest density of the SVMP deletion (the MS population in supplementary fig. S14A, Supplementary Material online) also showed among the highest average MYO copy numbers, yet we found no evidence for an association between these SVs. These loci appear to be evolving independently (Fig. 4c). The SVMP deletion was primarily detected in the western periphery of the species’ range and gradually declined in frequency eastward, becoming undetectable east of the Aucilla River in our sampling. MYO SVs had no well-defined west-to-east gradient, but instead decreased toward the southern edge of the range (supplementary fig. S14, Supplementary Material online). Previous work in *C. adamanteus* using neutral data from these same individuals (Fig. 4b; Margres et al. 2019) identified three genetically-distinct populations: one predominantly east and south of the Suwannee River, one predominantly west of the Suwannee River, and a distinct island population. MYO SVs were present in both western and eastern populations, with deletions being more frequent in the eastern population south of the Suwannee River (Margres et al. 2015b, 2017a). Given that the SVMP deletion was unique to the western population, our current sampling indicated that the Suwannee River may also be a phylogeographic barrier for the SVMP deletion; we do note that our current sampling does not indicate the presence of the SVMP deletion near the river (∼100 km west), and dense sampling along the contact zone would be needed to determine if the Suwannee River is a barrier to both neutral and putatively adaptive alleles. Nevertheless, the Suwannee River is thought to be a suture zone for numerous Florida lineages (Bert 1986), including *C. adamanteus* (Margres et al. 2015b, 2019). The SV in the SVMP array is a single large deletion that likely originated a single time in the western population, whereas the SVs involving the MYO genes comprise several deletion and/or duplication events that may have originated multiple times independently across the range, predate the phylogeographic split at the Suwannee River, and/or be shared through gene flow across the river (i.e. leaky barrier). The distinct genomic architectures of these loci may also partially explain their independent segregation and different geographic patterns. In contrast to SVMP loci (Fig. 2), the MYO loci are located in a genomic region of ma-2 that appears to be predisposed to intergenic rearrangements (Fig. 2) with a high density of TEs, which may promote copy-number variation. This region also harbors other gene families known to be highly duplicated, such as chemosensory and immunoglobulin genes (Hogan et al. 2021). Where examined in other rattlesnake species, MYO expression levels have nearly always been found to be highly variable within species (Bober et al. 1988; Oguiura et al. 2009; Gopalan et al. 2022), providing further evidence that this genomic region may experience higher rates of SV generation than others. We found that, in general, heterozygous SVs in the *C. adamanteus* genome occurred primarily in intergenic regions and were associated with enrichment of repetitive sequences and TEs. We also detected TEs flanking the region deleted in the SVMP tandem array, corroborating previous findings suggesting that TEs can directly affect snake venom variation (Dowell et al. 2016; Giorgianni et al. 2020; Perry et al. 2022). Structural variants differentiating haplotypes have been observed in numerous genomes from a diversity of eukaryotic species (Armstrong et al. 2022; Toh et al. 2022; Barros et al. 2023; Chang et al. 2023; Han et al. 2023; Qi et al. 2023; Zhao et al. 2023a), and these SVs were also found to be enriched in intergenic and repetitive regions (Casacuberta and González 2013; Serrato-Capuchina and Matute 2018).

Our detection of the SVMP deletion was contingent on fortuitously selecting a rare genotype in the region from which we sampled our genome animal. Given that this discovery resulted from a sample size of one (or two if counting haplotypes), additional genome sequencing from throughout the range is likely to reveal substantial genetic novelty that may affect management decisions for this species. *C. adamanteus* is a charismatic and emblematic species of the southeastern US coastal plain with a range substantially diminished from its historical extent as a result of human persecution and habitat loss (Means 2017). Given the numerous examples of large-scale venom-related expression differences within and between snake species with unknown genetic origins, including for *C. adamanteus* (Margres et al. 2016b, 2017b; Smith et al. 2023), we expect SVs to be a major source of this phenotypic variation and a critical component of functional genetic variation within species. These patterns of functional genetic variation should be incorporated into conservation and species-management decision-making processes, such as those currently ongoing for *C. adamanteus*. Previous work in *C. adamanteus* using neutral data from these same individuals (Fig. 4b; Margres et al. 2019) failed to detect any neutral population structure west of the Suwannee River. We found a major segregating SV in this region with a pronounced longitudinal frequency gradient, highlighting how the inclusion of functional genetic variation can refine our view of optimal conservation strategies. Not only does the western periphery of the range harbor a genetically and phenotypically unique population of *C. adamanteus*, the presence of this unique phenotype may indicate a novel ecology for the species in this region. High-contiguity genomes from multiple individuals from throughout the range will be necessary for a comprehensive accounting of all forms of genetic variation. Genomic approaches have long been used to identify cryptic species (Hinojosa et al. 2019; Christmas et al. 2021); high-accuracy, long-read sequencing technologies are now facilitating the identification of previously cryptic forms of genetic variation within species with implications for both species management as well as fundamental evolutionary processes.

## Materials and Methods

### Genome Sequencing

The PacBio HiFi data were described previously (Hogan et al. 2024). Briefly, data were generated using genomic DNA from an adult female (DRR0105). The resulting data comprised 3,910,111 reads with an average read length of 15.0 Kb and a total of 58,702,723,931 bp (>36× coverage). We used cutadapt version 4.1 (Martin 2011) to remove reads containing adapters and kraken2 version 2.1.2 (Wood et al. 2019) to remove human or bacterial contaminants.

A blood sample from the same individual (DRR0105) was used to construct an Hi-C library, following the protocol for nucleated blood cells for the Arima High Coverage Hi-C Kit (Arima Genomics) to crosslink DNA and generate the proximity-ligated DNA. We then constructed the final Hi-C library using the proximity-ligated DNA and the Arima High Coverage Hi-C Library Preparation Kit (Arima Genomics) following the manufacturer’s instructions. The Hi-C library was sequenced using the NovaSeq 6,000 platform (Illumina) with paired-end reads layout (2×150 bp) at the Florida State University College of Medicine Translational Science Laboratory. Sequencing yielded 269,208,645 paired-end reads. We used trim_galore! to trim adapters and to remove low-quality reads (−q 25) and reads shorter than 75 bp (--length 75), which returned a final dataset consisting of 265,684,197 paired-end reads (total of 79,218,703,363 bp; >49× coverage).

### Genome Assembly

The HiFi long reads and paired-end Hi-C short reads were provided to hifiasm version 0.16.1 (Cheng et al. 2021, 2022) to generate the primary and paired haplotype-resolved assembly contig graphs with default parameters. The contigs of the primary assembly were used as reference to map the Hi-C reads using Chromap v0.2.3 (Zhang et al. 2021a) and to scaffold using YaHS version 1.2a.2 (Zhou et al. 2023b). We then used Juicer version 1.6 (Durand et al. 2016) to manually review the scaffolded genome following the standard DNA Genome Assembly Cookbook instructions (https://aidenlab.org/assembly/manual_180322.pdf). The haplotype-resolved assemblies were generated using RagTag version 2.1.0 (Alonge et al. 2022) with each haplotype contig as a query and the primary chromosome-level assembly as a reference. Genome assembly statistics, for primary and both haplotypes, were obtained using Inspector version 1.0.1 (Chen et al. 2021), which also calculates a QV score to measure putative errors in the assembly, and the completeness was assessed using BUSCO version 5.2.2 (Waterhouse et al. 2018) with the Tetrapoda gene set (odb10; total of 5,310 genes). We also verified assembly quality using VerityMap version 1.0 (Bzikadze et al. 2022), which allows accurate mapping of long-reads to determine details about heterozygous and error-prone assembled regions. We characterized the identity of chromosomes performing BLAST searches against a set of chromosome-specific markers (NCBI accessions SAMN00177542 and SAMN00152474) of snakes (Matsubara et al. 2006) and the chromosome-level assembly of *C. viridis* (Schield et al. 2019). We also confirmed identities of sex chromosomes based on male–female read coverage ratio mapping whole-genome sequencing data of male and female individuals as previously described (Hogan et al. 2021). The mitochondrial genome was assembled using the long-read mode of MITGARD version 1.1 (Nachtigall et al. 2021a) with the HiFi reads and the *C. adamanteus* mitogenome as reference (NC_041524.1). The assembled mitogenome was annotated using MitoZ version 3.6 (Meng et al. 2019), followed by manual verification.

### Genome Annotation

We performed genome annotation on the primary chromosome-level assembly. We annotated repetitive regions and TEs using RepeatModeler2 and RepeatMasker. We used the RepeatModeler2 version 2.0.1 (Flynn et al. 2020) to generate a *de novo* species-specific repetitive-sequence and TE library. We split the library into “known” and “unknown” sets as output by RepeatModeler2. The “unknown” set was classified using DeepTE version 1.0 (Yan et al. 2020) with the model designed for metazoans. To remove false-positive repetitive elements, we removed any sequence classified as “NonTE” using TERL version 1.0 (da Cruz et al. 2021). Then, the species-specific TE library (i.e. the “known” set and the “unknown” re-classified set) was merged to a curated TE library designed for snakes (Castoe et al. 2013), and the final TE library was used to perform the repetitive annotation using RepeatMasker version 4.1.1 (https://www.repeatmasker.org/). The divergence between the individual TE copies versus their consensus sequences based on CpG-adjusted Kimura distance was estimated using RepeatMasker built-in scripts. We searched for telomeric sequences at chromosome terminals using tidk-search version 0.2.0 (Brown et al. 2023) using the conserved vertebrate telomeric repeat sequence TTAGGG.

Gene annotation was performed using the funannotate pipeline (Palmer and Stajich 2017), which integrates several *ab initio* gene predictors (i.e. AUGUSTUS, SNAP, and GeneMark-ES) to build gene models and uses transcript and protein evidence to generate the final annotation set. We used available transcriptomic data from several tissues from males and females of *C. adamanteus* (supplementary table S4, Supplementary Material online) as transcript evidence and the protein sequences available for the Tetrapoda clade in Uniprot and NCBI databases as protein evidence. We also performed the functional annotation step using InterProScan5 version 5.54 (Jones et al. 2014). Due to the high number of proteins predicted by funannotate, we compared the predicted proteins to the proteins annotated in the genomes of mouse, chicken, green anole, Central bearded dragon, Komodo dragon, Common wall lizard, Mainland tiger snake, and Eastern brown snake available in the ENSEMBL database (downloaded August 2023) using DIAMOND version 2.1.9 (Buchfink et al. 2021) with high stringency (parameters set to be more sensitive, minimum coverage of 50%, and *e*-value <0.001) to confirm conserved and confident predicted proteins. To annotate toxins, we used ToxCodAn-Genome version 1.0 (Nachtigall et al. 2024) with default parameters and followed their guide to ensure a confident toxin annotation set (Nachtigall 2023). Briefly, the genome individual venom-gland transcriptomic data were assembled and annotated using ToxCodAn version 1.0 (Nachtigall et al. 2021b) with default parameters to generate a species-specific toxin database. The species-specific and the Viperidae toxin databases were used as database sources to annotate the toxins in the genome using ToxCodAn-Genome version 1.0 (Nachtigall et al. 2024). We then merged the toxin and nontoxin annotations to generate a final annotation set. The final annotation set obtained in the primary assembly was lifted to the haplotype-resolved assemblies using liftoff version 1.6.3 (Shumate and Salzberg 2021).

### Comparative Analysis

We compared haplotypes at the sequence level using syri version 1.6.3 (Goel et al. 2019) and at the gene level using genespace version 1.3.0 (Lovell et al. 2022). We also compared the assemblies on a smaller scale by aligning the genomic region containing the multiloci toxin families (i.e. SVMP, SVSP, PLA2, MYO, and CTL) to check for differences in these regions between haplotypes and primary assemblies. Genomic alignments were performed using BLAST (blastn) with an identity percentage threshold set to 95%. We then filtered results to keep alignments >10 Kb and plotted the alignments using ggplot2 in R.

To ensure that putative SVs detected between haplotypes were not assembly artifacts, we mapped the HiFi reads against each of the assemblies (i.e. primary and both haplotypes) using VerityMap, Inspector, and minimap2. We then analyzed read coverage and the VerityMap output in the toxin genomic regions of interest to check for error-prone regions. VerityMap maps long reads using a k-mer method and enables checking for possible errors and heterozygous sites in the assembly on the basis of the proportion of rare k-mers. To check whether multimapped reads could be influencing detection of assembly artifacts in those regions, we filtered the minimap2 output to only keep uniquely mapped and high-quality alignments by removing reads with MAPQ <30 using samtools. We also checked for collapsed regions in the highly duplicated toxin genomic regions using NucFreq version 0.1 (Vollger et al. 2019) and the mapping files output by VerityMap, Inspector, and minimap2. NucFreq checks for collapsed regions that may indicate assembly artifacts occurring in error-prone regions. We performed this additional step because analyzing the mapping status of the original reads along a genome assembly allows assessment of the overall assembly quality and can reveal putative assembly artifacts and error-prone regions (Li et al. 2023).

We performed a phylogenetic inference for the SVSP and SVMP toxin genes to better understand their relationships. We aligned their coding sequences using MAFFT version 7.450 (Rozewicki et al. 2019) with default parameters and searched for the maximum likelihood tree using IQTree version 1.6.12 (Nguyen et al. 2015) with parameters −  m TEST  −bb 1000  −alrt 1000. The final tree was adjusted using FigTree version 1.4.4 (https://github.com/rambaut/figtree/).

### Venom-gland Transcriptomic Analysis

We used previously published venom-gland transcriptomic data (supplementary table S4, Supplementary Material online) for 18 individuals of *C. adamanteus* (Hogan et al. 2024). Adapters and low-quality reads were removed using trim_galore! as previously described. Expression levels of annotated coding sequences were estimated using RSEM version 1.3.1 (Li and Dewey 2011) using Bowtie2 version 2.4.2 (Langmead and Salzberg 2012) as the aligner with the mismatch rate parameter set to 0.02.

### Exon-capture Data Analysis

We used a set of anchored data available for 139 individuals of *C. adamanteus* designed to sequence the exon of toxin genes and other probes as previously described (Margres et al. 2017a, 2019). These data comprise individuals sampled from throughout the species distribution and contain representatives of most *C. adamanteus* populations. Adapters and low-quality reads were trimmed using trim_galore! as previously described. The trimmed reads were mapped against the primary assembly using Bowtie2. We removed PCR duplicates and mapped reads with MAPQ <30 before calculating the average coverage of each SVMP paralog to genotype each individual as homozygous or heterozygous for the SVMP deletion observed in the haplotype-revolved assemblies. Specifically, we calculated the average of coverage for each SVMP gene in the SVMP array. Then, the individuals were genotyped as follows: (i) homozygous for the entire SVMP array (HomoRef), when all genes were presenting a similar average of coverage; (ii) heterozygous for the SVMP deletion (Het), when genes in the SVMP deletion were presenting half of average of coverage when compared with the SVMP genes not located in the SVMP deletion; and (iii) homozygous for the SVMP deletion (HomoDel), when genes in the SVMP deletion presented an average of coverage <10% of the other genes in the SVMP array.

To estimate the copy number of MYO genes in each sample, we mapped reads as described above, but the multimapped reads were kept due to the high similarity of MYO genes (i.e. we did not remove mapped reads with MAPQ <30). We then used the coverage of exon 2 and exon 3 from MYO genes to calculate the average of coverage, as previously performed (Margres et al. 2017a), and compared it with the average coverage of 10 nontoxin genes available in the probe set and located on the macrochromosome 2 as well (i.e. ATPSynLipid-1, ATPase-lys70, CD63, Calreticulin, DAZ-2, GADD45, Glutaredoxin-1, Leptin-1, PDI, and Nexin-2).

### Venom Reversed-Phase High-Performance Liquid Chromatography

To visualize the compositional effects of the SVMP deletion, we performed reversed-phase high-performance liquid chromatography (RP-HPLC) for two individuals genotyped as homozygotes for each haplotype (i.e. two individuals homozygous for the complete SVMP array and two individuals homozygous for the six-paralog SVMP deletion). We performed RP-HPLC analysis on pairs of individuals collected in close geographic proximity. RP-HPLC was performed and analyzed as previously described (Margres et al. 2015a).

### Venom Mass Spectrometry

To generate a genotype–phenotype map and verify the toxin expression proteomically, we performed mass spectrometry on whole venom samples from genotyped individuals for the SVMP deletion. Proteomics data were generated and analyzed following (Hofmann et al. 2018). See Supplementary material online for details.

### Permits and Protocols

The specimen used for genome sequencing was collected under the Florida USA permits LSSC-13-00004A, LSSC-13-00004B, and LSSC-13-00004C. All animal procedures were performed under active IACUC protocols: Florida State University protocols 0924, 1230, 1333, 1529, and 1836.

## Supplementary Material

msaf058_Supplementary_Data

## Data Availability

A list with all datasets used in the present study are available in supplementary table S4, Supplementary Material online. The genome assembly and the Hi-C data generated in the present study are available in the NCBI under the project number PRJNA868880. In addition, the assembled genome and annotations are available in the figshare database (https://figshare.com/projects/Eastern_diamondback_rattlesnake_Crotalus_adamanteus_-_haplotype-resolved_genome_assembly/200614). All code and commands used in this study are available on GitHub (https://github.com/pedronachtigall/Cadamanteus_SV).
