# Supplementary material for: A Segregating Structural Variant Defines Novel Venom Phenotypes in the Eastern Diamondback Rattlesnake
Source: Mol Biol Evol. 2025 Mar 18;42(4):msaf058. doi: 10.1093/molbev/msaf058 (PMC11965796; doi:10.1093/molbev/msaf058)
Supplement: msaf058_Supplementary_Data [file msaf058_supplementary_data.pdf]

Supplementary Information

Supplementary Materials and Methods

High-molecular-weight DNA isolation and PacBio HiFi sequencing

As previously reported (Hogan *et al.* 2024), blood was extracted from the caudal vein of an adult female of *Crotalus adamanteus* (DRR0105; 125 cm snout-to-vent length) from the Apalachicola National Forest in the Florida panhandle and stored in 95% ethanol at $-80°C$ until use. High-molecular-weight (HMW) genomic DNA (gDNA) was extracted by using a pipette-free protocol as previously described (Margres *et al.* 2021). HMW DNA was used to construct the PacBio HiFi sequencing libraries with the SMRTbell Express Template Prep Kit 2.0 following the manufacturer's protocol. Separate HMW DNA extractions from the same animal were sent to two different sequencing facilities: the Clemson Center for Human Genetics and the University of Delaware DNA Sequencing and Genotyping Center. At each facility, one flowcell of data was generated on a PacBio Sequel II instrument using the HiFi sequencing protocol. The combined sequencing runs resulted in 3,910,111 reads with an average read length of 15.0 Kb and a total of 58,702,723,931 bp (>36-fold coverage).

Venom proteomics

We first quantified venom samples using the Qubit Protein Assay kit with a Qubit 1.0 Fluorometer (Thermo Fisher Scientific). For each sample, we digested $\sim$5 μg of whole venom using the Calbiochem ProteoExtract All-in-One Trypsin Digestion Kit (Merck, Darmstadt, Germany) according to the manufacturer's instructions and using LC/MS grade solvents, yielding $\sim$4.3 μg of digested venom protein after digestion. Samples were dried using a SpeedVac at 25℃ for 1 hour and stored at -20℃ until use. The trypsin-digested samples were used as input for the mass spectometry analysis.

Mass spectrometry (MS) was performed at the Translational Science Laboratory in the College of Medicine at Florida State University. An externally calibrated Thermo Scientific Orbitrap Exploris 480 mass spectrometer (high-resolution electrospray tandem MS) was used in conjunction with Thermo Scientific EASY-nLC 1200 system. Trypsin-digested sample (1 μl) was loaded onto the trap column (Acclaim PepMap 100, 100 μm $\times$ 2 cm, nanoViper; Thermo Scientific). The flow rate was set to 300 nl/min for separation on the analytical column (Acclaim pepmap RSLC, 75 μM $\times$ 15 cm, nanoViper; Thermo Scientific). Mobile phase A was composed of 99.9% H_2O containing 0.1% formic acid and mobile phase B was composed of 95% acetonitrile, 4.9% H_2O, and 0.1% formic acid. A 60-minute linear gradient from 3% to 45% mobile phase B was performed. The LC eluent was directly nanosprayed into the Exploris 480 mass spectrometer. During chromatographic separation, the Exploris 480 was operated in a data-dependent mode under the direct control of the Thermo Excalibur 4.4.16.14 (Thermo Scientific). The MS data were acquired using the following parameters: 30 data-dependent collisional-induced-dissociation (CID) MS/MS scans per full scan (350 to 1700 m/z) at 120,000 resolution in profile mode. MS2 were acquired in centroid mode at 15,000 resolution. Ions with a single charge, more than 7 charges, or unassigned charges were excluded. A 15-second dynamic exclusion window was used. All measurements were performed at room temperature, and three technical replicates were run for each sample. The raw files were analyzed using Thermo Proteome Discoverer (version 2.5.0.400) software package with SequestHT search node using the protein-coding genes annotated in the genome (i.e., toxins and nontoxins) as the database and the Percolator peptide validator. The SequestHT search parameters used were: enzyme name = Trypsin, maximum missed cleavage = 2, minimum peptide length = 6, maximum peptide length = 144, maximum delta Cn = 0.05, precursor mass tolerance = 10 ppm, fragment mass tolerance = 0.02 Da, dynamic modifications, carbamidome-thyl + 57.021 Da(C) and oxidation + 15.995 Da(M). The resulting .msf files were further analyzed by the proteome validator software Scaffold v5.3.0 (Portland, OR, USA). We accepted protein identities based on a 1.0% false discovery rate (FDR) using the Scaffold Local FDR algorithm and a minimum of one unique peptide in at least one sample. The quantification of proteins was based on normalized Exclusive Unique Spectral Counts (EUSC), corresponding to the number of spectra attributed to proteotypic peptides of a given protein entry present in the database.

Supplementary Figures

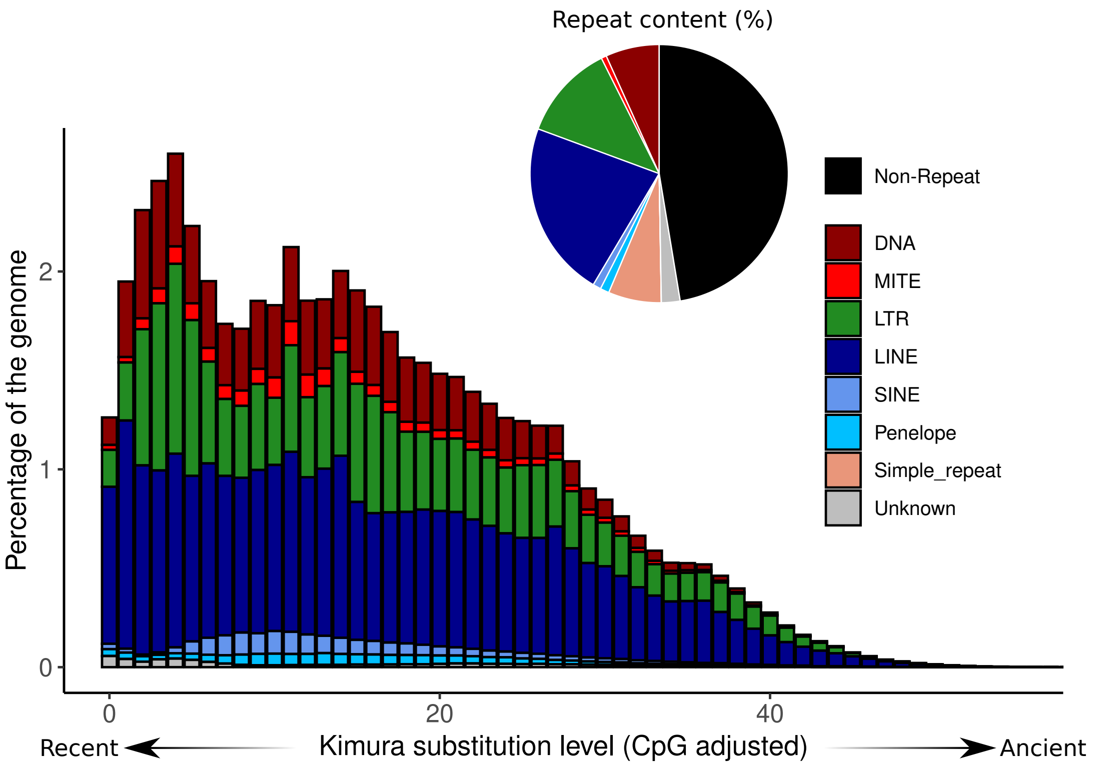

Fig. S1. Repeat landscape for the *Crotalus adamanteus* genome. The pie chart shows the percent abundance of each repeat class in the genome. The barplot shows the TE accumulation history in the genome. The *x*-axis is the CpG-adjusted Kimura distance from the consensus sequence in the TE library, whereas the *y*-axis is the percentage of TE occupancy in the genome. Copies in the left region of the plot have not diverged substantially from the consensus sequence for that class and likely represent recent copies, whereas sequences on the right exhibit greater divergence from the consensus and likely correspond to more ancient copies. Overall, the repeat landscape is characterized by recent bursts of LINE elements. Abbreviations: DNA—DNA transposable element, MITE—Miniature inverted-repeat transposable element, LTR—Long terminal repeat element, LINE—Long interspersed nuclear element, SINE—Short interspersed nuclear element.

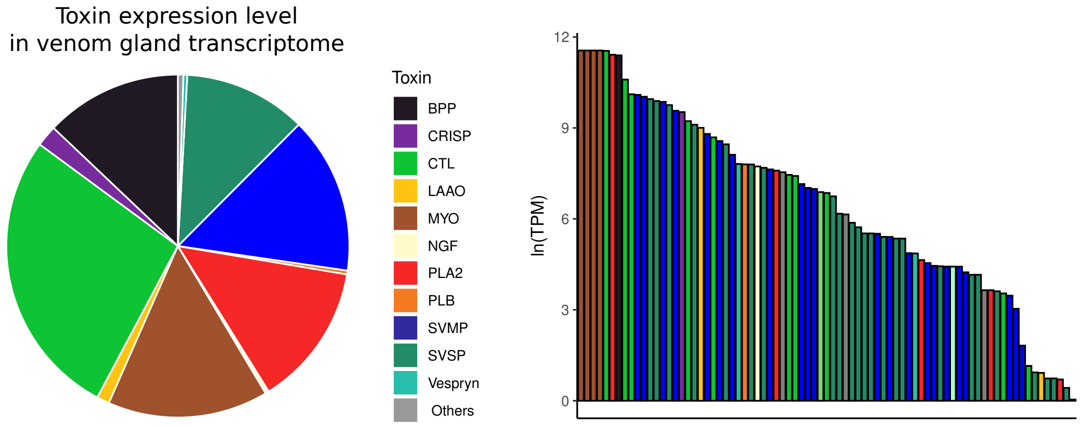

Fig. S2. Pie chart and barplot showing the toxin expression level in the venom-gland transcriptomic data obtained for the individual used to sequence the genome (DRR0105; Table S4). MYO, SVMP, PLA2, and CTLs are major components of the venom-gland transcriptome. Abbreviations: BPP–Bradykinin-potentiating peptides, CRISP—Cysteine-rich secretory proteins, CTL—C-type lectins, KUN—Kunitz-type proteinase inhibitor, LAAO—L-amino acid oxidase, MYO—Crotamin, NGF—Nerve growth factor, NUC—Ecto 5' nucleotidase, PDE—Phosphodiesterase, PLA2—Phospholipase A_2, PLB—Phospholipase B, SVMP—Snake venom metalloproteinase, SVSP—Snake venom serine protease.

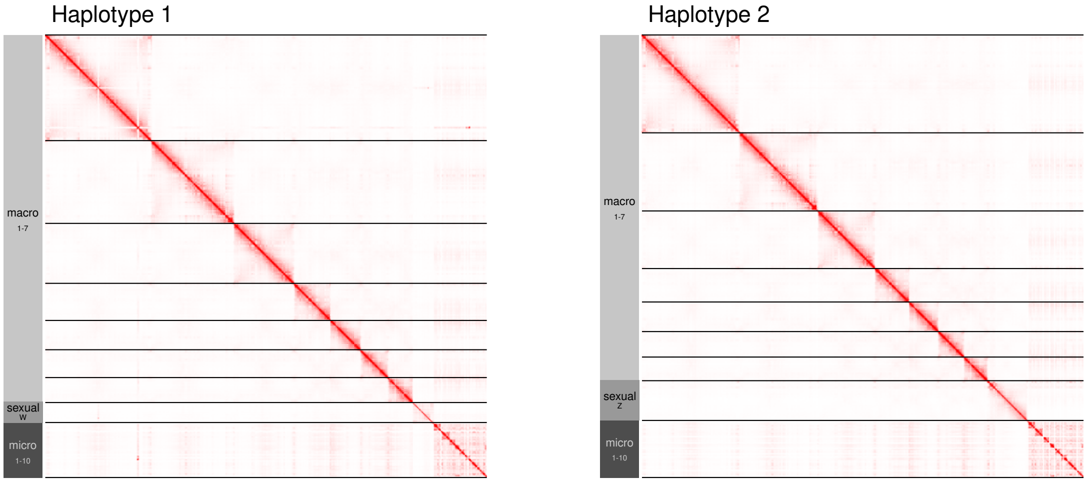

Fig. S3. Hi-C interactions among 18 chromosomes within each haplotype assembled for *C. adamanteus*. Darker color indicates stronger interactions. The patterns observed suggest that both haplotypes were correctly assembled.

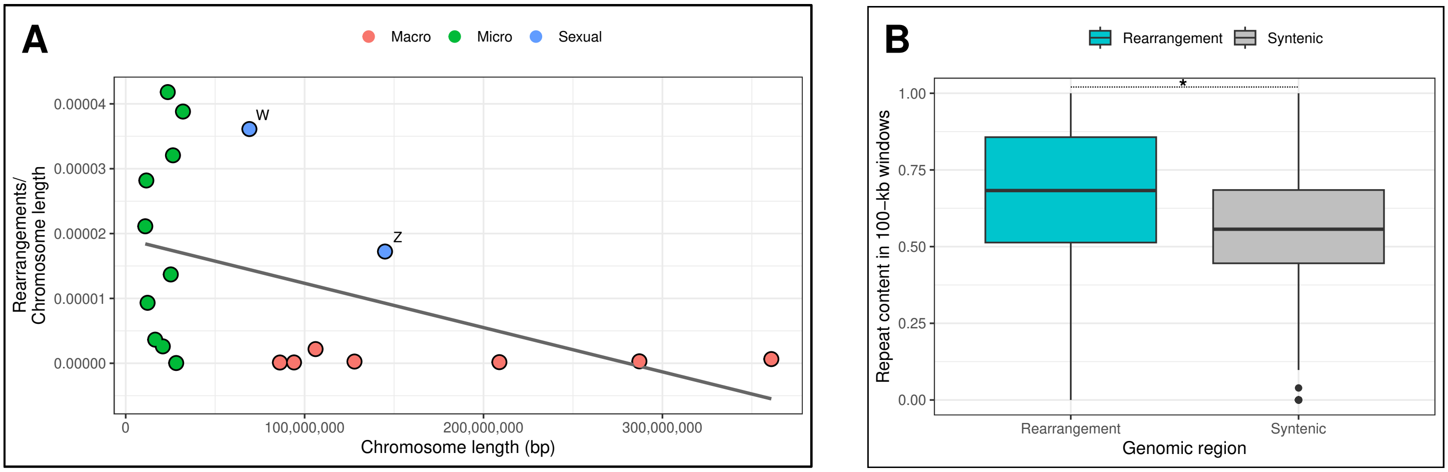

Fig. S4. Number of rearrangements per chromosome length and repetitive content in rearranged and syntenic genomic regions between haplotypes. (A) The number of rearrangements in microchromosomes and sex chromosomes were higher than for autosomes and macrochromosomes. The line indicates the correlation between number of rearrangements and chromosome length. (B) The content of repetitive sequences in rearrangements are higher than in syntenic regions. This analysis was based on 1,793 sliding windows of rearrangement regions and 2,334 sliding windows of syntenic regions. The asterisk indicates a statistically significant difference in repeat content between rearrangements and syntenic regions using the Wilcoxon rank sum test (W = 2,760,648, $p < 2.2 \times 10^{-16}$).

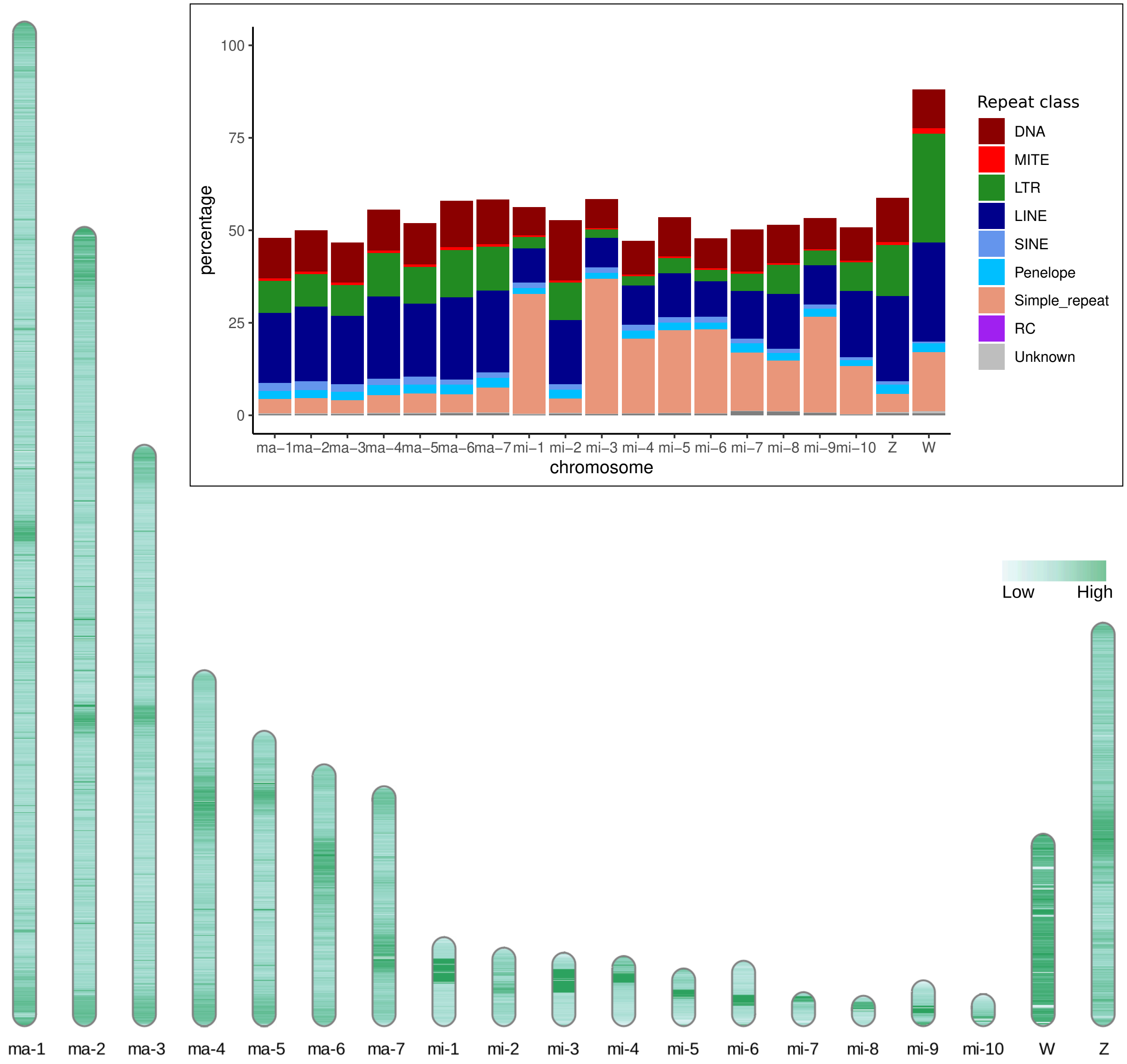

Fig. S5. Repetitive sequence distribution and percentage of each repeat class annotated in each chromosome. The karyotype plot shows the distribution of high and low repetitive regions among the genome in a 100-Kb window, whereas the barplot shows the percentage of repetitive classes in each chromosome. This data reveals that the W chromosome presents a higher proportion of repetitive sequences distributed along its entire chromosome when compared to Z and other chromosomes. Abbreviations: DNA—DNA transposable element, MITE—Miniature inverted-repeat transposable element, LTR—Long terminal repeat element, LINE—Long interspersed nuclear element, SINE—Short interspersed nuclear element.

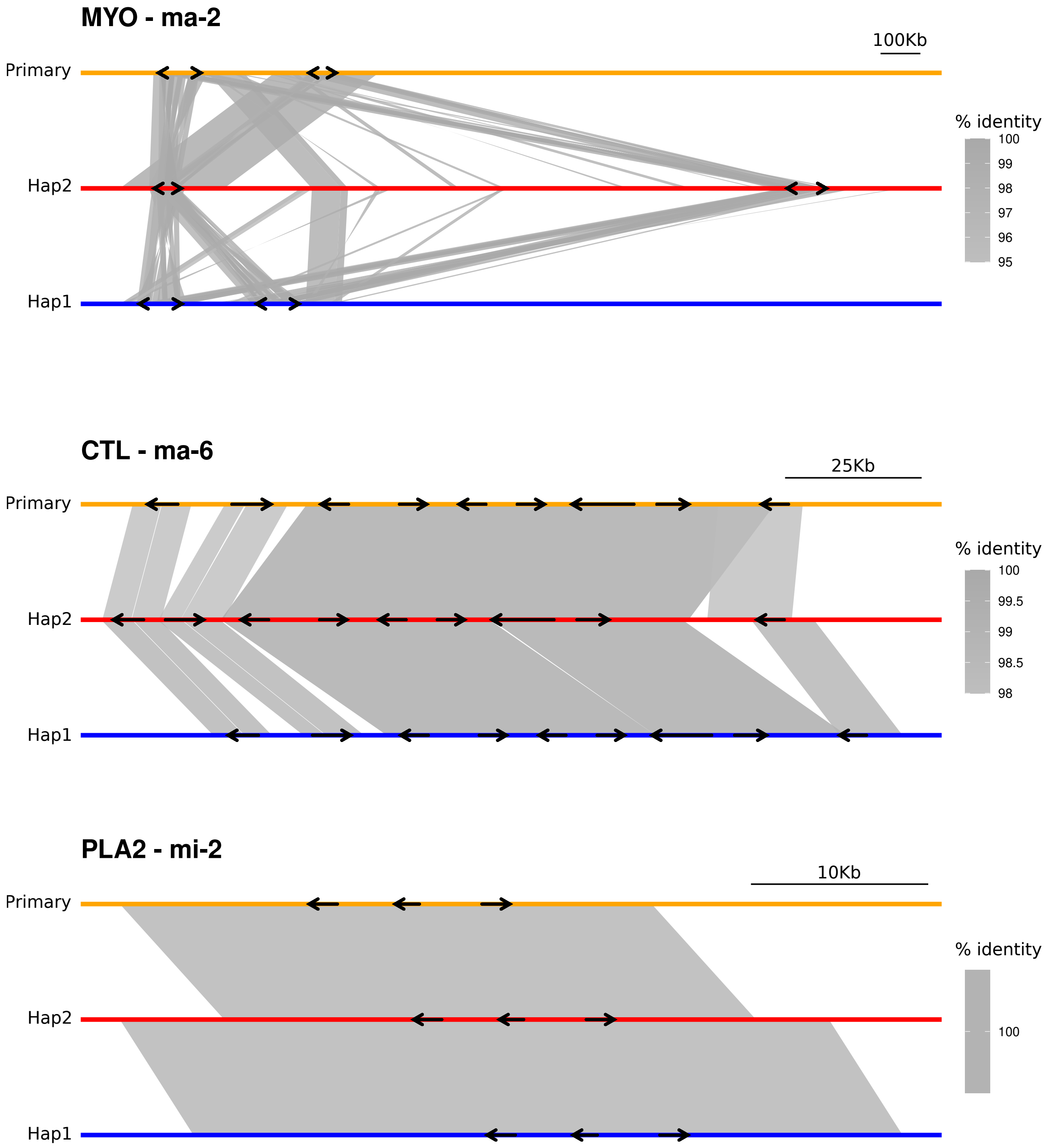

Fig. S6. Riparian plots showing the genomic alignments of the MYO, PLA2, CTL toxin arrays between primary and both haplotypes. Black arrows represent each toxin gene in that specific family. The grey shaded areas represent the percentage identity between alignments obtained through BLAST search, which was filtered to sizes >10Kb and percentage identity >95%. The black line is the scale bar to represent a size of 100Kb, 25Kb, and 10Kb for MYO, CTL, and PLA2, respectively. This data indicates that these toxin families present a similar pattern of toxin gene content, despite some rearrangements observed in intergenic regions of MYO and CTL arrays. Abbreviations: BPP—Bradykinin-potentiating peptide, CRISP—Cysteine-rich secretory protein, CTL—C-type lectin, HYAL—Hyaluronidase, KUN—Kunitz-type protease inhibitor, LAAO—L-amino acid oxidase, MYO—Myotoxin/crotamin, NGF—Nerve growth factor, NUC—Nucleotidase, PDE—Phosphodiesterase, PLA2—Phospholipase A_2, PLB—Phospholipase B, SVMP—Snake venom metalloproteinase, SVSP—Snake venom serine proteinase.

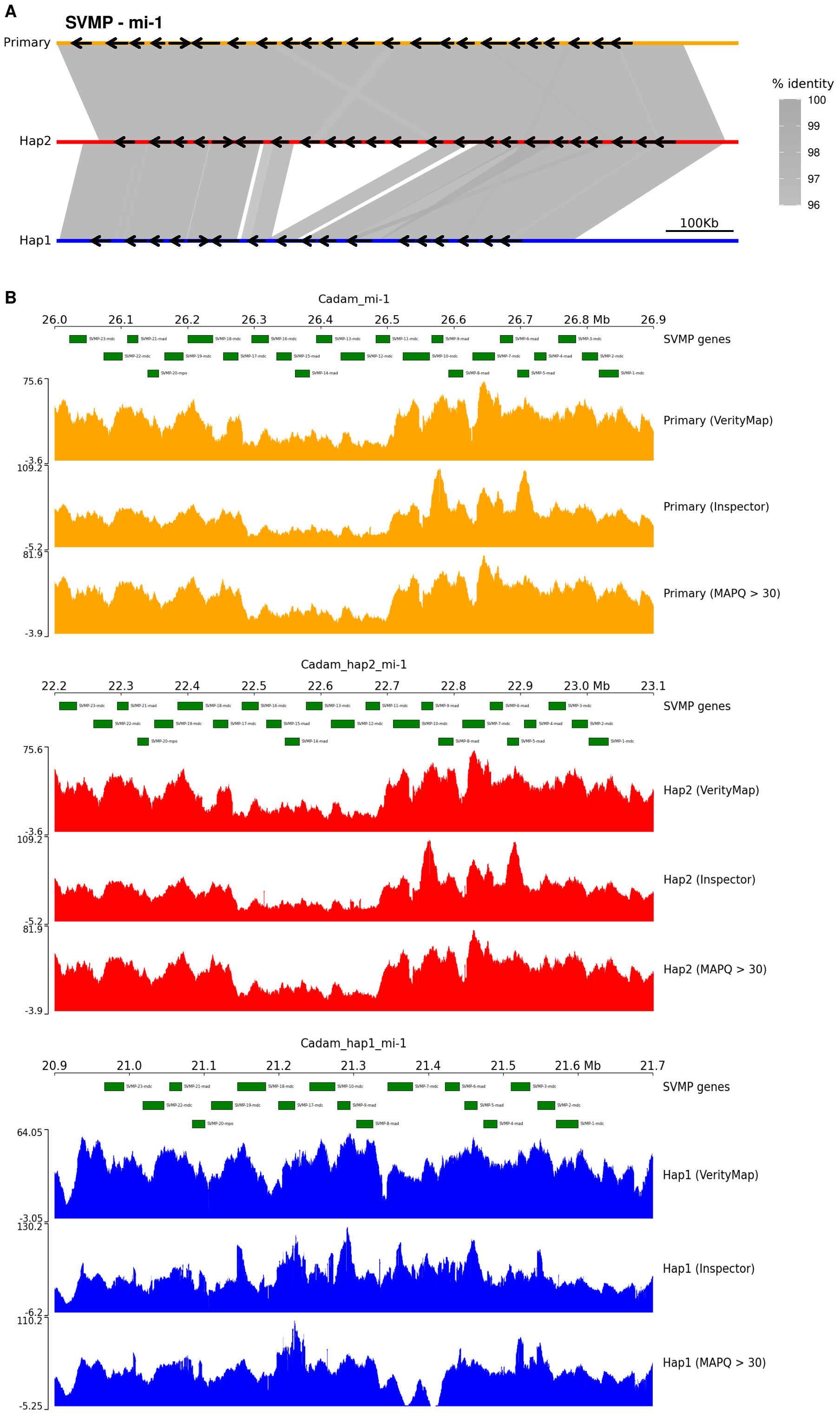

Fig. S7. Genome alignment of SVMP array and read coverage of the PacBio HiFi data in each assembly. (A) Riparian plot showing the alignments of the toxin family SVMP between primary and both haplotypes. Black arrows represent each toxin gene in that specific family. The grey shaded areas represent the percentage identity between alignments obtained through BLAST search, which was filtered to keep alignments with size >10Kb and percentage identity >95%). The black line is the scale bar to represent a size of 100Kb. The alignment shows high synteny between primary and haplotype 2 and a deletion of 6 SVMP genes in haplotype 1 when compared to haplotype 2 and primary assemblies. (B) SVMP read coverage in primary and both haplotypes using the PacBio HiFi data. Mapping coverage obtained using VerityMap to check for error-prone regions, Inspector that calculated the QV score, and Minimap2 filtered to keep only MAPQ >30 (i.e., removing multi-mapped reads). Green boxes at the top represent the toxin genes identified in each assembly. In the coverage plots, we noticed a similar read coverage in breakpoints and flanking regions, which indicates no error-prone regions in all assemblies. This pattern was confirmed by the VerityMap output, which revealed no error-prone regions.

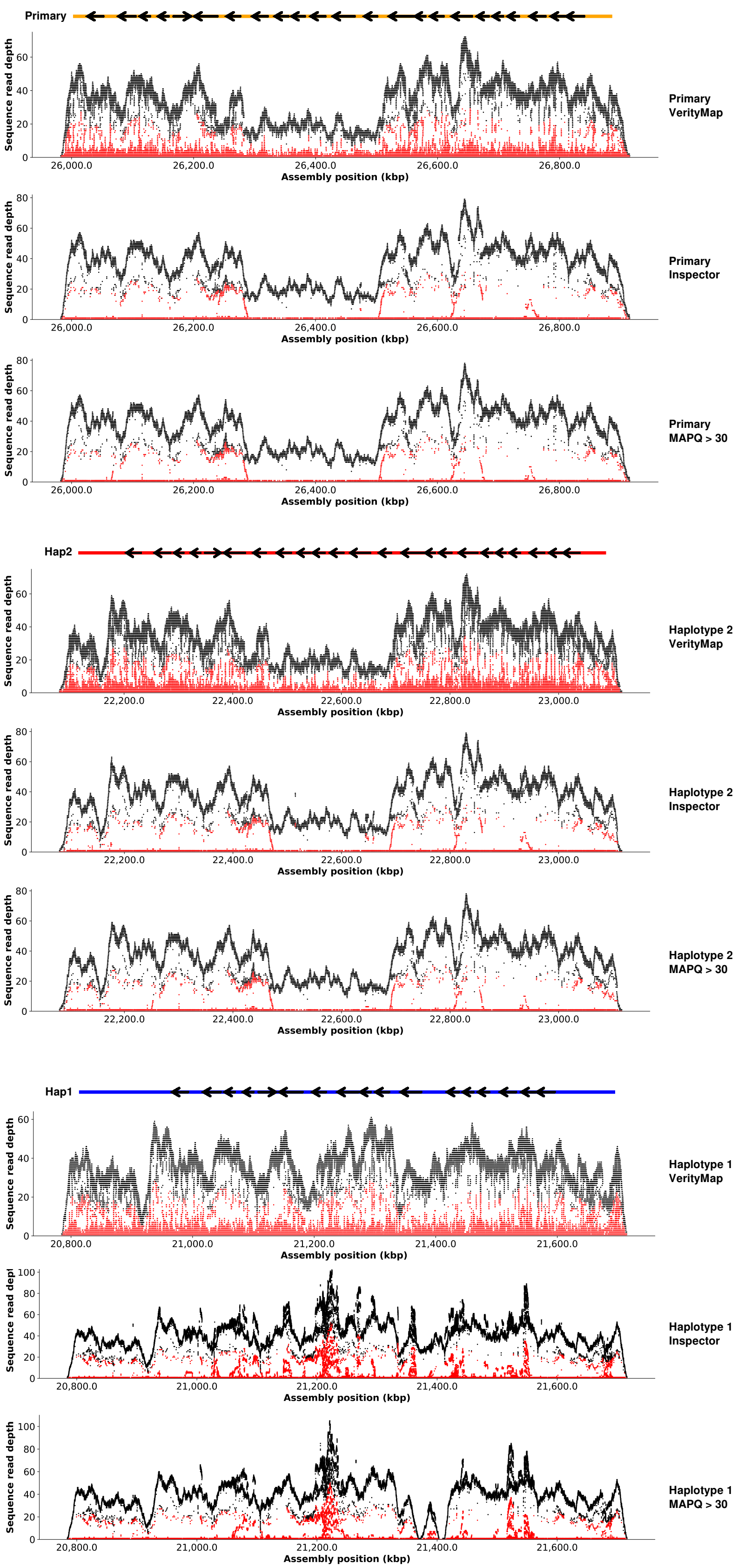

Fig. S8. NucFreq plot (Vollger *et al.* 2019) of the SVMP array using the PacBio HiFi data obtained for each assembly (from top to bottom: primary, haplotype 2, and haplotype 1). The NucFreq plot for the SVMP region revealed no evidence of collapsed regions in any of the assemblies, which indicates no errors in the assemblies (Vollger *et al.* 2019). Of note, the slight increased read coverage observed in the breakpoint of the SVMP deletion in hap1 in both Inspector and filtered minimap2 is expected once we used the reads comprising both haplotypes to map against the assemblies.

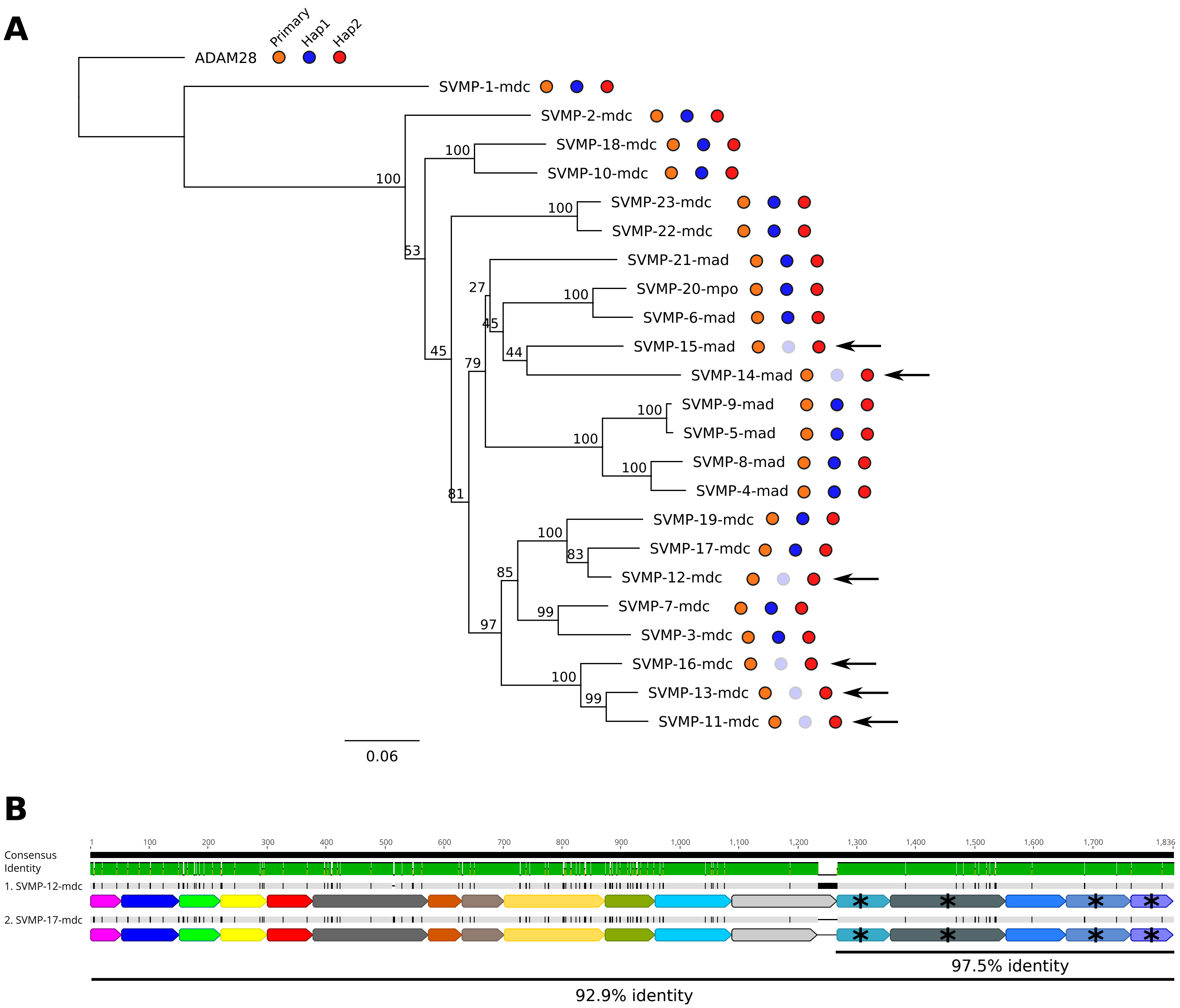

Fig. S9. SVMP gene tree and alignment of two SVMP genes. (A) SVMP gene tree showing the relationship of SVMP genes identified in *C. adamanteus* genome. The colored circles represent the assemblies where the genes were identified. Transparent circles mean the gene is absent in that specific assembly. Orange color represents the primary, blue color represents the haplotype 1, and red color represents the haplotype 2. The support values of bootstrap are shown at tree nodes. The SVMP genes with copy number variation among assemblies are indicated with an arrow. (B) CDS alignment of SVMP-12-mdc and SVMP-17-mdc to show the high similarity between genes. The percent identity for each site is represented by a consensus bar at the top. The 17 exons within the CDSs are represented by arrows and color-coded by their position from first to last for each sequence. There is an increase in the identity of the last five exons sequences as indicated by the black lines at the bottom. The "*" indicates exons with read coverage in the anchored data of homozygotes for the SVMP deletion.

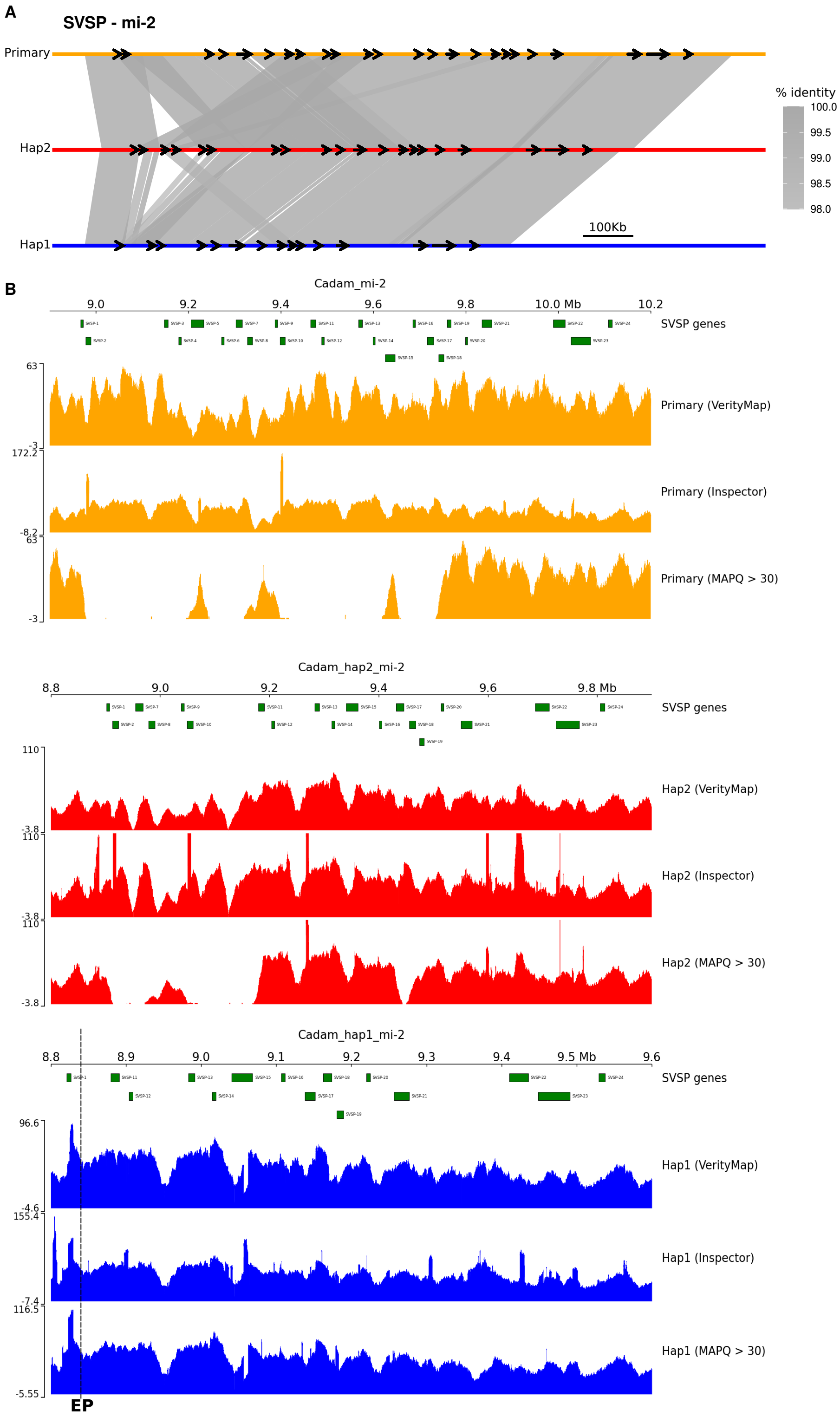

Fig. S10. Genomic alignment of SVSP array and read coverage of the PacBio HiFi data in each assembly. (A) Riparian plot showing the genomic alignments of the toxin family SVSP between primary and both haplotypes. Black arrows represent each toxin gene in that specific family. The grey shaded areas represent the percentage identity between alignments obtained through BLAST search, which was filtered to keep alignments with size >10Kb and percentage identity >95%). The black line is the scale bar to represent a size of 100Kb. (B) SVSP read coverage in primary and both haplotypes using the PacBio HiFi data. Mapping coverage obtained using VerityMap to check for error-prone regions (i.e., vertical dashed lines indicates with an "EP" coloured in black), Inspector that calculated the QV score, and Minimap2 filtering to keep only MAPQ >30 (i.e., removing multi-mapped reads). Green boxes at the represent the toxin genes identified in each assembly. In the coverage plots, we noticed a similar read coverage along the entire SVSP array in VerityMap output in all assemblies; however, VerityMap identified an error-prone region in the breakpoint of Hap1 as indicated by the "EP" mark. The Inspector output and the minimap2 filtered output revealed that the primary and Hap2 assemblies presents regions with high increased and decrease of read coverage, which includes the breakpoints and their flanking regions. The read coverage pattern observed indicates that the SVSP genomic region is hard to resolve and assemble due to the presence of error-prone regions. Further experiments are necessary to confirm this assembled region.

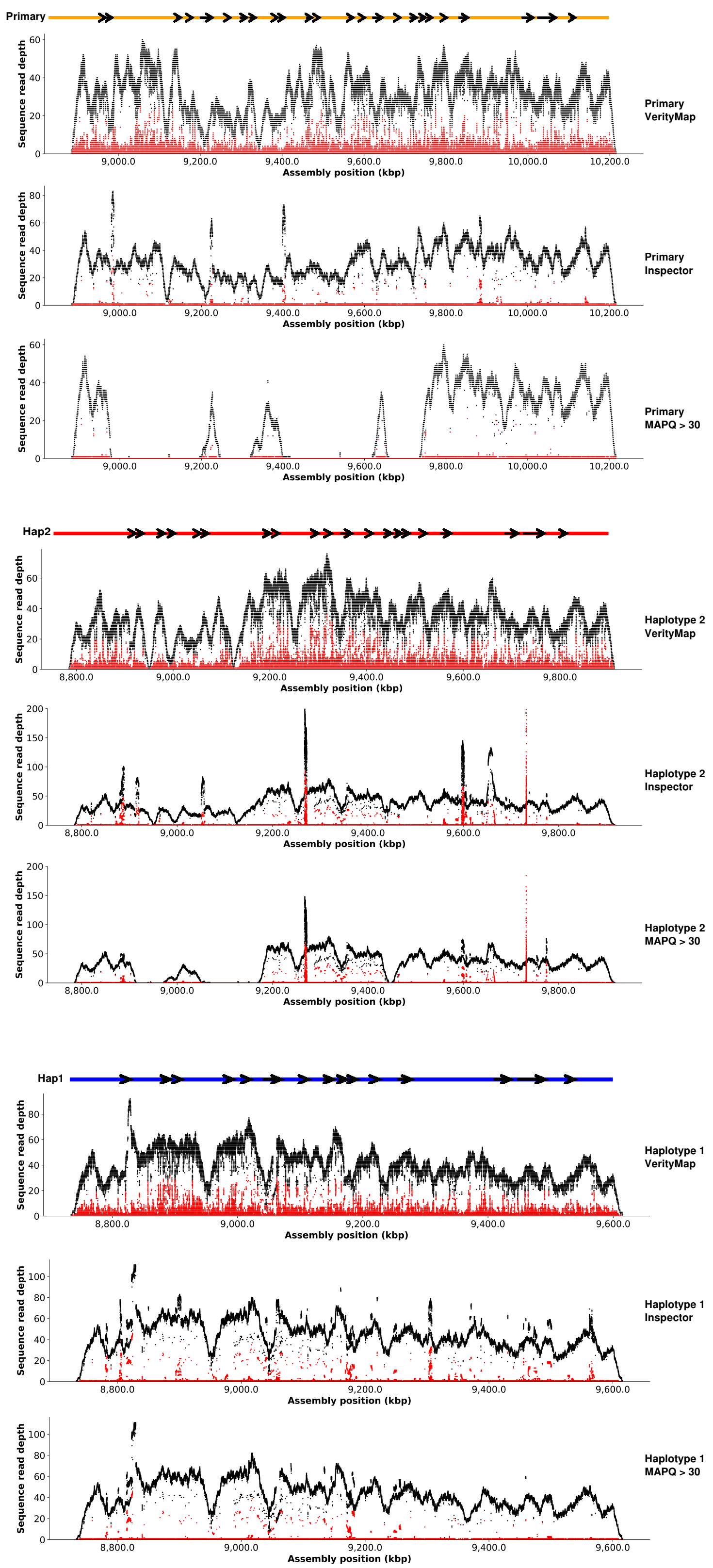

Fig. S11. NucFreq plot (Vollger *et al.* 2019) of the SVSP array using the PacBio HiFi data obtained for each assembly (from top to bottom: primary, haplotype 2, and haplotype 1). The NucFreq plot for the SVSP region revealed evidence of collapsed regions in this highly duplicated genomic region in all assemblies using the output from Inspector and filtered minimap2. Those collapsed regions are mainly located at the breakpoints of the differences observed among haplotypes. In summary, this analysis revealed that this genomic region is hard to resolve and assemble due to the presence of error-prone regions. Further experiments are necessary to confirm this assembled region.

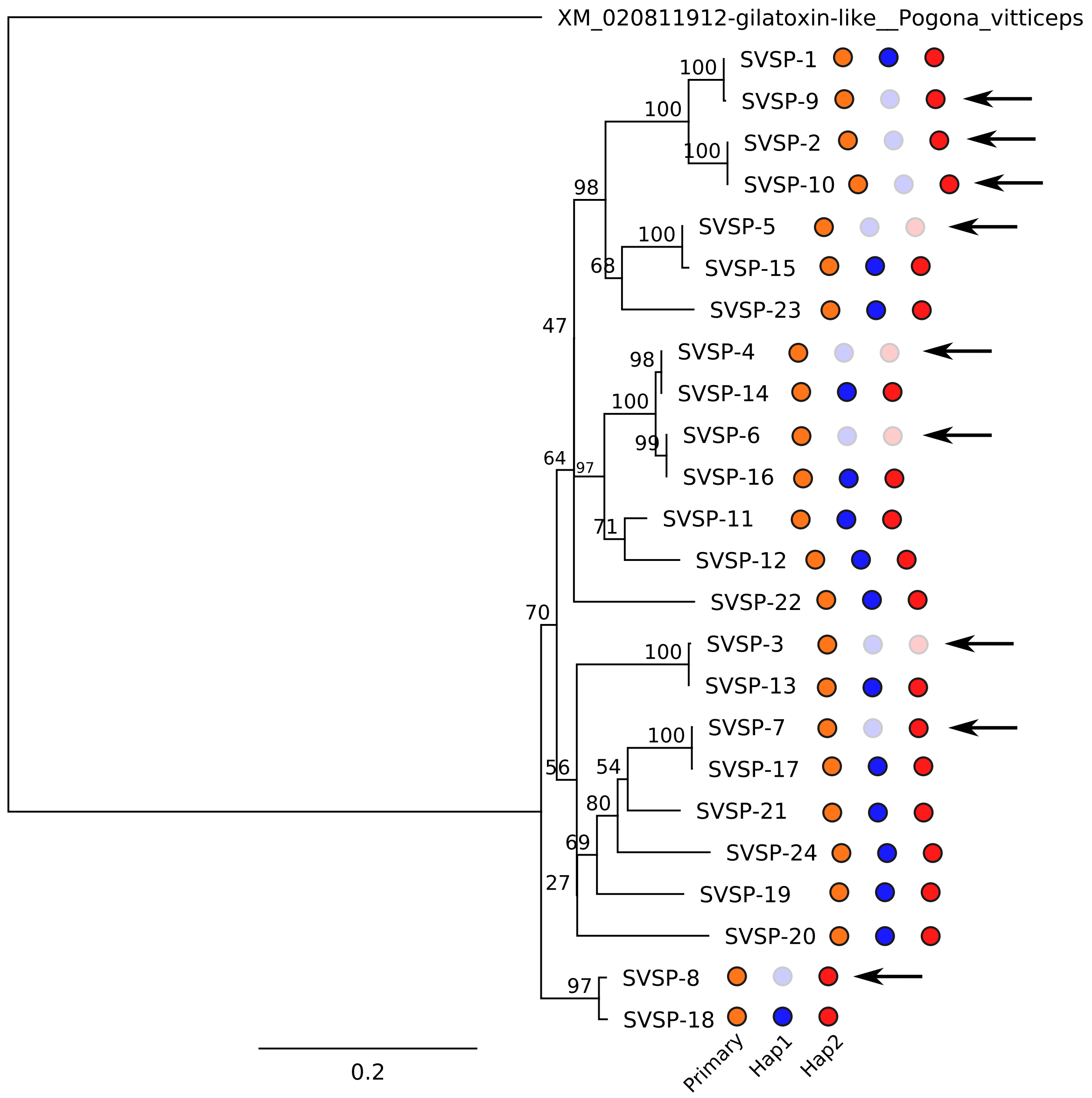

Fig. S12. SVSP gene tree showing the relationship of SVSP genes identified in *C. adamanteus* genome. The colored circles represent the assemblies where the genes were identified. Transparent circles mean the gene is absent in that specific assembly. Orange color represents the primary, blue color represents the haplotype 1, and red color represents the haplotype 2. The support values of bootstrap are shown at tree nodes. The SVSP genes with copy number variation among assemblies are indicated with an arrow.

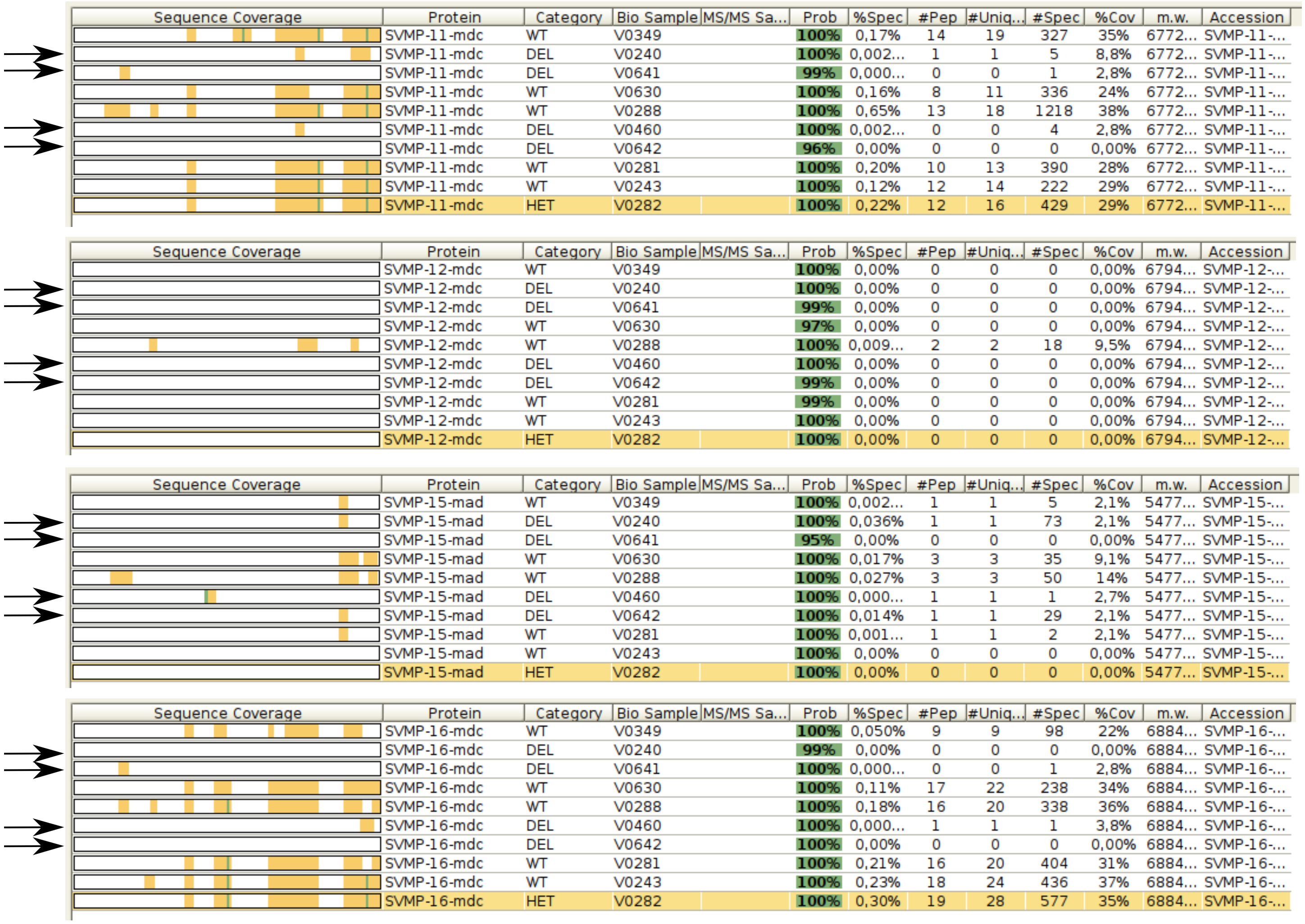

Sequence Coverage	Protein	Category	Bio Sample	MS/MS Sa...	Prob	%Spec	#Pep	#Uniq...	#Spec	%Cov	m.w.	Accession
	SVMP-11-mdc	WT	V0349		100%	0,17%	14	19	327	35%	6772...	SVMP-11-...
	SVMP-11-mdc	DEL	V0240		100%	0,002...	1	1	5	8,8%	6772...	SVMP-11-...
	SVMP-11-mdc	DEL	V0641		99%	0,000...	0	0	1	2,8%	6772...	SVMP-11-...
	SVMP-11-mdc	WT	V0630		100%	0,16%	8	11	336	24%	6772...	SVMP-11-...
	SVMP-11-mdc	WT	V0288		100%	0,65%	13	18	1218	38%	6772...	SVMP-11-...
	SVMP-11-mdc	DEL	V0460		100%	0,002...	0	0	4	2,8%	6772...	SVMP-11-...
	SVMP-11-mdc	DEL	V0642		96%	0,00%	0	0	0	0,00%	6772...	SVMP-11-...
	SVMP-11-mdc	WT	V0281		100%	0,20%	10	13	390	28%	6772...	SVMP-11-...
	SVMP-11-mdc	WT	V0243		100%	0,12%	12	14	222	29%	6772...	SVMP-11-...
	SVMP-11-mdc	HET	V0282		100%	0,22%	12	16	429	29%	6772...	SVMP-11-...

Sequence Coverage	Protein	Category	Bio Sample	MS/MS Sa...	Prob	%Spec	#Pep	#Uniq...	#Spec	%Cov	m.w.	Accession
	SVMP-12-mdc	WT	V0349		100%	0,00%	0	0	0	0,00%	6794...	SVMP-12-...
	SVMP-12-mdc	DEL	V0240		100%	0,00%	0	0	0	0,00%	6794...	SVMP-12-...
	SVMP-12-mdc	DEL	V0641		99%	0,00%	0	0	0	0,00%	6794...	SVMP-12-...
	SVMP-12-mdc	WT	V0630		97%	0,00%	0	0	0	0,00%	6794...	SVMP-12-...
	SVMP-12-mdc	WT	V0288		100%	0,009...	2	2	18	9,5%	6794...	SVMP-12-...
	SVMP-12-mdc	DEL	V0460		100%	0,00%	0	0	0	0,00%	6794...	SVMP-12-...
	SVMP-12-mdc	DEL	V0642		99%	0,00%	0	0	0	0,00%	6794...	SVMP-12-...
	SVMP-12-mdc	WT	V0281		99%	0,00%	0	0	0	0,00%	6794...	SVMP-12-...
	SVMP-12-mdc	WT	V0243		100%	0,00%	0	0	0	0,00%	6794...	SVMP-12-...
	SVMP-12-mdc	HET	V0282		100%	0,00%	0	0	0	0,00%	6794...	SVMP-12-...

Sequence Coverage	Protein	Category	Bio Sample	MS/MS Sa...	Prob	%Spec	#Pep	#Uniq...	#Spec	%Cov	m.w.	Accession
	SVMP-15-mad	WT	V0349		100%	0,002...	1	1	5	2,1%	5477...	SVMP-15-...
	SVMP-15-mad	DEL	V0240		100%	0,036%	1	1	73	2,1%	5477...	SVMP-15-...
	SVMP-15-mad	DEL	V0641		95%	0,00%	0	0	0	0,00%	5477...	SVMP-15-...
	SVMP-15-mad	WT	V0630		100%	0,017%	3	3	35	9,1%	5477...	SVMP-15-...
	SVMP-15-mad	WT	V0288		100%	0,027%	3	3	50	14%	5477...	SVMP-15-...
	SVMP-15-mad	DEL	V0460		100%	0,000...	1	1	1	2,7%	5477...	SVMP-15-...
	SVMP-15-mad	DEL	V0642		100%	0,014%	1	1	29	2,1%	5477...	SVMP-15-...
	SVMP-15-mad	WT	V0281		100%	0,001...	1	1	2	2,1%	5477...	SVMP-15-...
	SVMP-15-mad	WT	V0243		100%	0,00%	0	0	0	0,00%	5477...	SVMP-15-...
	SVMP-15-mad	HET	V0282		100%	0,00%	0	0	0	0,00%	5477...	SVMP-15-...

Sequence Coverage	Protein	Category	Bio Sample	MS/MS Sa...	Prob	%Spec	#Pep	#Uniq...	#Spec	%Cov	m.w.	Accession
	SVMP-16-mdc	WT	V0349		100%	0,050%	9	9	98	22%	6884...	SVMP-16-...
	SVMP-16-mdc	DEL	V0240		99%	0,00%	0	0	0	0,00%	6884...	SVMP-16-...
	SVMP-16-mdc	DEL	V0641		100%	0,000...	0	0	1	2,8%	6884...	SVMP-16-...
	SVMP-16-mdc	WT	V0630		100%	0,11%	17	22	238	34%	6884...	SVMP-16-...
	SVMP-16-mdc	WT	V0288		100%	0,18%	16	20	338	36%	6884...	SVMP-16-...
	SVMP-16-mdc	DEL	V0460		100%	0,000...	1	1	1	3,8%	6884...	SVMP-16-...
	SVMP-16-mdc	DEL	V0642		100%	0,00%	0	0	0	0,00%	6884...	SVMP-16-...
	SVMP-16-mdc	WT	V0281		100%	0,21%	16	20	404	31%	6884...	SVMP-16-...
	SVMP-16-mdc	WT	V0243		100%	0,23%	18	24	436	37%	6884...	SVMP-16-...
	SVMP-16-mdc	HET	V0282		100%	0,30%	19	28	577	35%	6884...	SVMP-16-...

Fig. S13. Proteomics data from venom samples of genotyped individuals for the SVMP deletion. The data is shown only for SVMPs located at the deletion for visualizing purposes. The arrows indicate the homozygotes for the SVMP deletion (DEL). The other samples were homozygotes for the entire array (WT) and heterozygote (HET). The coverage of deleted SVMP within those samples indicates that those low number of unique peptides and exclusive spectra counts observed may likely represent artifacts from proteomics approaches. These results confirm the absence of those SVMPs in the venom of homozygotes for the SVMP deletion (DEL).

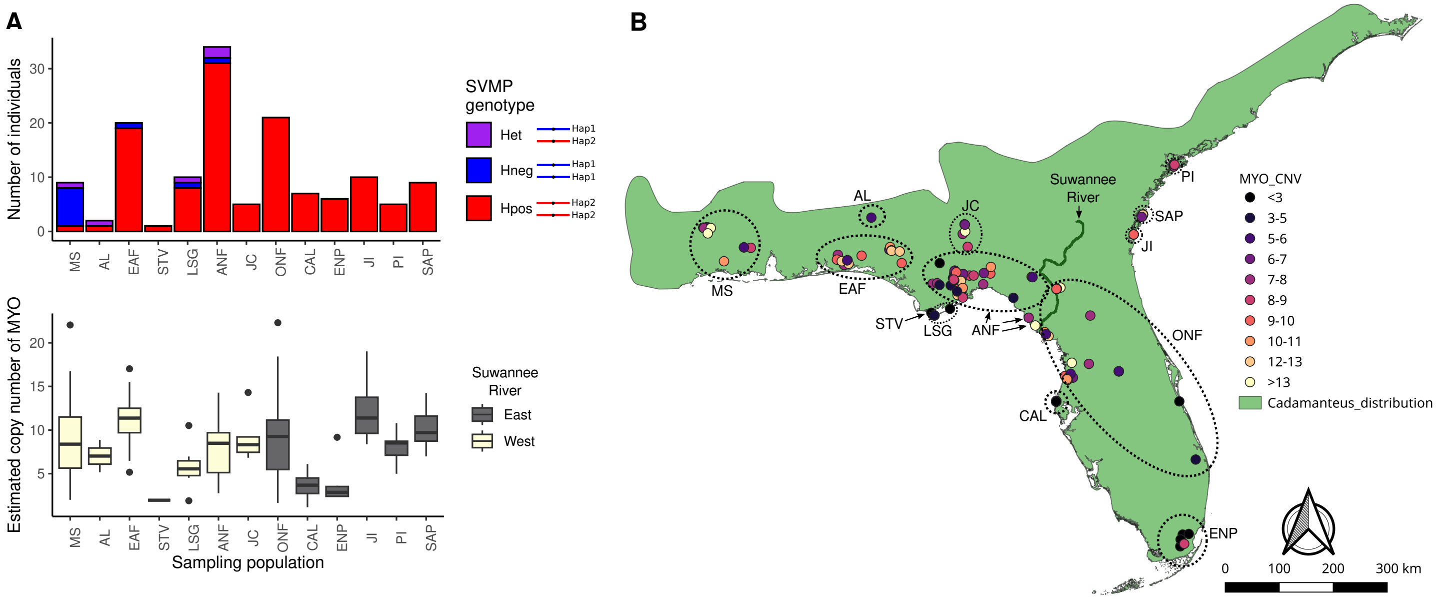

Fig. S14. Myotoxin/crotamine (MYO) copy number variation among individuals analyzed in the present study. (A) Barplot at the top showing the number of individuals sampled within each population and the SVMP gentoypes, where the boxplot at the bottom show the estimate copy number of MYO genes among individuals of the same populations. The boxplot is colored considering their sampling sites as west or east of the Suwannee River, which may represent a physical barrier for the SVMP deletion as previously discussed (Margres *et al.* 2017, 2019). (B) Map showing the geopgraphical distribution of samples with their estimate copy numbers of MYO. The populations are labeled as defined in both barplot and boxplot.

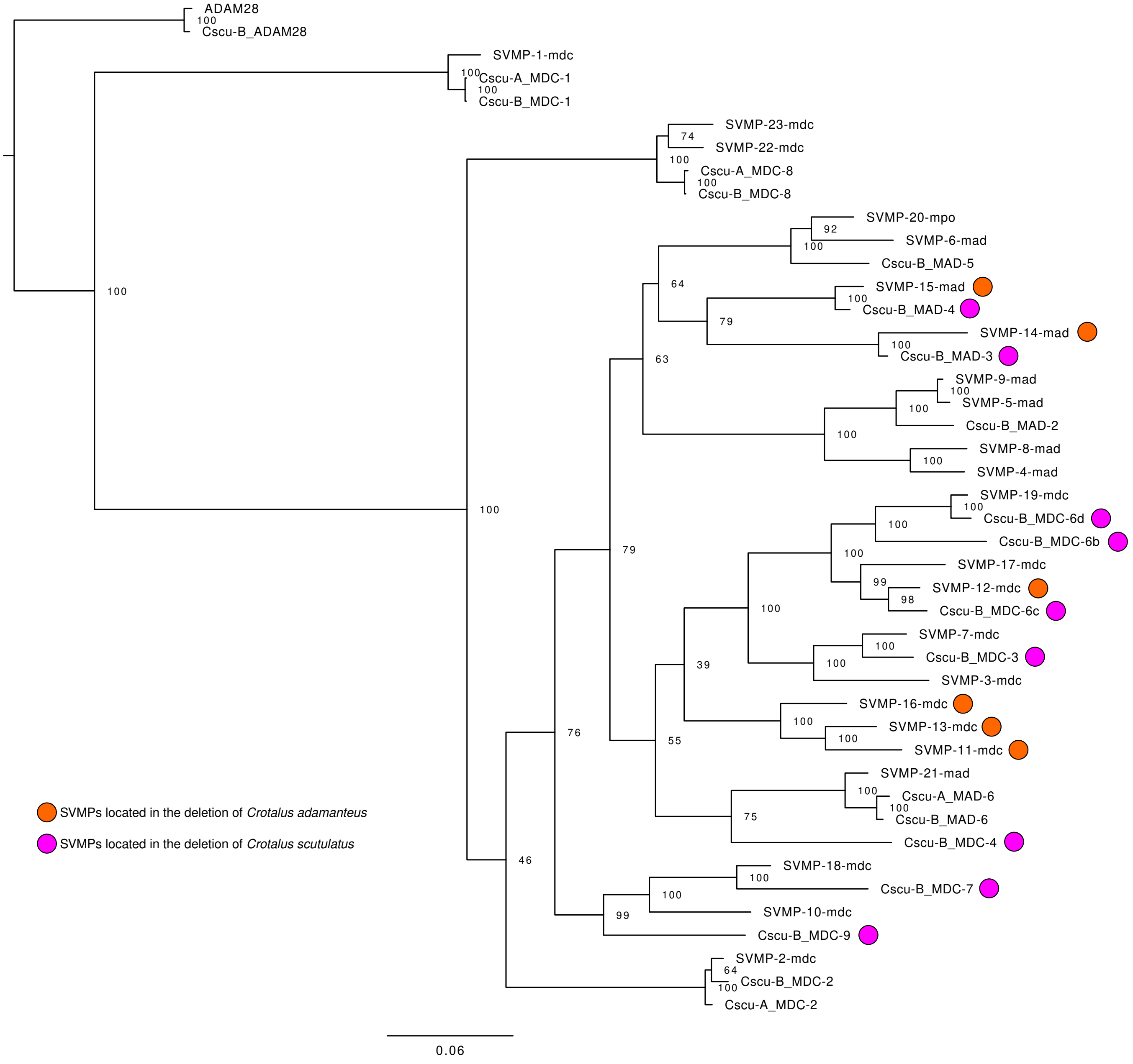

Fig. S15. Gene tree comparing the SVMPs in *C. adamanteus* to the SVMPs from both A and B haplotypes of *C. scutulatus*. SVMPs located in the deletions of both species are highlighted with orange and pink circles for *C. adamanteus* and *C. scutulatus*, respectively. Bootstrap support values are shown at tree nodes. Sequences from *C. scutulatus* were obtained from Giorgianni *et al.* (2020). NCBI accession numbers: MT070613 for the A haplotype and MT032003 for the B haplotype.

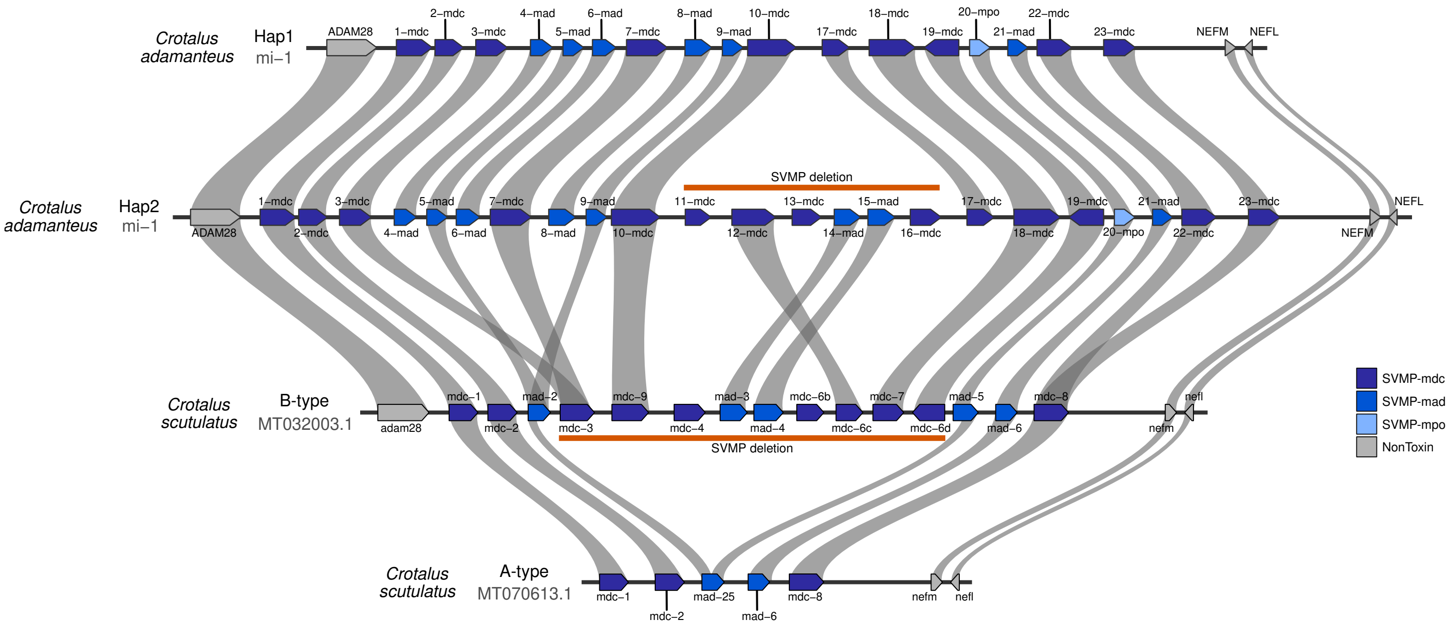

Fig. S16. Genomic alignment of SVMP loci for both haplotypes of *C. adamanteus* and *C. scutulatus*. The riparian plots are showing relationships of SVMPs based on the phylogenetic tree. The SVMPs in the deletions are indicated with orange lines for both species. Sequences from *C. scutulatus* were obtained from Giorgianni *et al.* (2020). NCBI accession numbers: MT070613 for the A haplotype and MT032003 for the B haplotype.

Supplementary Tables

Table S1. Summary of toxin gene annotation in primary and both haplotypes assembled for *C. adamanteus*.

		Primary			Haplotype 1			Haplotype 2		
Toxin	Chr	Start	End	Str	Start	End	Str	Start	End	Str
KUN-1	ma-1	63,795,270	63,827,434	+	63,639,567	63,677,464	+	63,135,736	63,167,900	+
CTL-10-lectin	ma-1	113,472,640	113,479,676	–	113,085,929	113,092,965	–	112,746,956	112,753,993	–
CRISP-1	ma-1	193,696,212	193,705,298	+	196,179,289	196,188,355	+	192,080,106	192,089,192	+
NUC-1	ma-1	240,037,486	240,071,472	+	242,433,636	242,467,622	+	238,459,484	238,493,494	+
PDE-1	ma-1	262,425,538	262,502,046	+	264,743,141	264,820,310	+	260,807,614	260,884,122	+
VEGF-A	ma-1	292,026,806	292,048,725	+	294,355,387	294,377,306	+	290,309,947	290,331,834	+
MYO-1	ma-2	397,195	398,443	–	848,872	850,120	–	436,357	437,605	–
MYO-2	ma-2	502,044	503,292	+	954,293	955,541	+	2,122,466	2,123,713	+
MYO-3	ma-2	774,919	776,167	–	1,143,525	1,144,773	–	2,025,732	2,026,980	–
MYO-4	ma-2	841,662	842,910	+	1,248,374	1,249,622	+	503,100	504,348	+
Vespryn-1	ma-2	26,454,646	26,458,695	+	26,283,664	26,287,713	+	27,354,616	27,358,640	+
LAAO-1	ma-2	26,779,255	26,801,317	–	26,608,273	26,630,335	–	27,685,727	27,712,835	–
LAAO-2	ma-2	26,943,443	26,967,555	+	26,772,461	26,796,573	+	27,838,991	27,863,103	+
NGF-1	ma-3	171,680,570	171,681,295	–	171,090,619	171,091,344	–	171,285,740	171,286,465	–
BPP-1	ma-5	101,947,072	101,948,099	+	100,711,702	100,712,735	+	101,602,839	101,603,866	+
HYAL-1	ma-6	21,648,959	21,658,346	–	21,729,496	21,738,883	–	21,880,694	21,890,081	–
PLB-1	ma-6	62,499,430	62,544,538	–	62,744,817	62,788,852	–	62,665,621	62,710,729	–
CTL-1-beta	ma-6	93,415,066	93,420,612	–	93,239,829	93,245,375	–	93,450,252	93,451,312	–
CTL-2-alpha	ma-6	93,430,503	93,437,641	+	93,255,266	93,262,237	+	93,452,256	93,453,390	+
CTL-3-beta	ma-6	93,446,815	93,451,846	–	93,271,418	93,276,450	–	93,462,564	93,467,595	–
CTL-4-alpha	ma-6	93,461,184	93,466,145	+	93,285,783	93,290,760	+	93,476,933	93,481,894	+
CTL-5-beta	ma-6	93,472,098	93,477,005	–	93,296,718	93,301,641	–	93,487,847	93,492,754	–
CTL-6-alpha	ma-6	93,482,728	93,487,698	+	93,307,366	93,312,355	+	93,498,477	93,503,447	+
CTL-7-beta	ma-6	93,492,756	93,504,086	–	93,317,415	93,328,332	–	93,508,505	93,519,835	–
CTL-8-alpha	ma-6	93,508,288	93,514,101	+	93,332,534	93,338,353	+	93,524,037	93,529,850	+
CTL-9-beta	ma-6	93,527,457	93,532,383	–	93,351,739	93,356,664	–	93,556,985	93,561,983	–
SVMP-23-mdc	mi-1	26,022,353	26,048,096	–	20,966,842	20,992,579	–	22,207,244	22,232,987	–
SVMP-22-mdc	mi-1	26,073,845	26,101,964	–	21,018,327	21,046,457	–	22,258,736	22,286,855	–
SVMP-21-mad	mi-1	26,109,244	26,125,625	–	21,053,738	21,070,108	–	22,294,135	22,310,516	–
SVMP-20-mpo	mi-1	26,139,800	26,156,513	–	21,084,283	21,100,995	–	22,324,691	22,341,404	–
SVMP-19-mdc	mi-1	26,165,228	26,193,328	+	21,109,829	21,137,921	+	22,350,119	22,378,219	+
SVMP-18-mdc	mi-1	26,200,152	26,237,725	–	21,144,744	21,182,255	–	22,385,043	22,422,618	–
SVMP-17-mdc	mi-1	26,253,633	26,275,668	–	21,199,081	21,221,092	–	22,438,526	22,460,561	–
SVMP-16-mdc	mi-1	26,296,415	26,321,514	–	NA	NA	NA	22,481,308	22,506,407	–
SVMP-15-mad	mi-1	26,333,751	26,355,736	–	NA	NA	NA	22,518,644	22,540,629	–
SVMP-14-mad	mi-1	26,361,609	26,382,990	–	NA	NA	NA	22,546,502	22,567,883	–
SVMP-13-mdc	mi-1	26,393,370	26,417,044	–	NA	NA	NA	22,578,263	22,601,937	–
SVMP-12-mdc	mi-1	26,430,291	26,465,736	–	NA	NA	NA	22,615,184	22,650,629	–
SVMP-11-mdc	mi-1	26,482,543	26,503,464	–	NA	NA	NA	22,667,436	22,688,357	–
SVMP-10-mdc	mi-1	26,523,705	26,563,155	–	21,241,041	21,274,849	–	22,708,598	22,748,048	–
SVMP-9-mad	mi-1	26,566,612	26,583,597	–	21,278,307	21,295,294	–	22,751,505	22,768,490	–
SVMP-8-mad	mi-1	26,592,000	26,613,839	–	21,303,698	21,325,541	–	22,776,893	22,798,732	–
SVMP-7-mdc	mi-1	26,627,953	26,661,086	–	21,345,562	21,378,668	–	22,812,846	22,845,980	–
SVMP-6-mad	mi-1	26,669,444	26,688,626	–	21,422,304	21,441,486	–	22,854,338	22,873,520	–
SVMP-5-mad	mi-1	26,695,593	26,712,446	–	21,448,453	21,465,306	–	22,880,487	22,897,340	–
SVMP-4-mad	mi-1	26,720,663	26,738,762	–	21,473,523	21,491,622	–	22,905,557	22,923,656	–
SVMP-3-mdc	mi-1	26,757,303	26,782,993	–	21,510,163	21,535,853	–	22,942,197	22,967,889	–
SVMP-2-mdc	mi-1	26,792,795	26,815,957	–	21,545,655	21,568,817	–	22,977,677	23,000,828	–
SVMP-1-mdc	mi-1	26,818,118	26,847,159	–	21,570,978	21,600,019	–	23,002,989	23,032,115	–
KUN-2	mi-2	170,094	175,545	+	164,769	170,235	+	170,096	175,547	+
SVSP-1	mi-2	8,967,357	8,972,866	+	8,821,310	8,826,819	+	8,901,853	8,907,362	+
SVSP-2	mi-2	8,978,179	8,989,250	+	NA	NA	NA	8,912,675	8,923,746	+
SVSP-3	mi-2	9,148,027	9,156,482	+	NA	NA	NA	NA	NA	NA
SVSP-4	mi-2	9,179,443	9,184,482	+	NA	NA	NA	NA	NA	NA
SVSP-5	mi-2	9,205,810	9,233,363	+	NA	NA	NA	NA	NA	NA
SVSP-6	mi-2	9,271,783	9,276,879	+	NA	NA	NA	NA	NA	NA
SVSP-7	mi-2	9,303,203	9,316,973	+	NA	NA	NA	8,954,722	8,968,492	+
SVSP-8	mi-2	9,327,300	9,338,558	+	NA	NA	NA	8,978,819	8,990,077	+
SVSP-9	mi-2	9,387,108	9,392,616	+	NA	NA	NA	9,038,627	9,044,135	+
SVSP-10	mi-2	9,397,929	9,408,998	+	NA	NA	NA	9,049,448	9,060,517	+
SVSP-11	mi-2	9,464,504	9,475,641	+	8,879,840	8,890,972	+	9,179,724	9,190,858	+
SVSP-12	mi-2	9,488,531	9,493,807	+	8,903,862	8,909,138	+	9,203,748	9,209,024	+
SVSP-13	mi-2	9,567,772	9,576,227	+	8,983,108	8,991,563	+	9,282,957	9,291,412	+
SVSP-14	mi-2	9,599,206	9,604,246	+	9,014,524	9,019,563	+	9,314,374	9,319,414	+
SVSP-15	mi-2	9,625,573	9,647,306	+	9,040,891	9,068,444	+	9,340,741	9,362,476	+
SVSP-16	mi-2	9,685,538	9,690,620	+	9,106,864	9,111,960	+	9,400,701	9,405,783	+
SVSP-17	mi-2	9,716,945	9,730,745	+	9,138,284	9,152,036	+	9,432,108	9,445,908	+

SVSP-18	mi-2	9,741,081	9,752,325	+	9,162,372	9,173,612	+	9,456,244	9,467,488	+
SVSP-19	mi-2	9,759,393	9,768,367	+	9,180,680	9,189,654	+	9,474,556	9,483,530	+
SVSP-20	mi-2	9,798,856	9,803,881	+	9,220,143	9,225,168	+	9,514,019	9,519,044	+
SVSP-21	mi-2	9,835,318	9,855,768	+	9,256,605	9,277,055	+	9,550,481	9,570,931	+
SVSP-22	mi-2	9,989,255	10,014,465	+	9,410,542	9,435,752	+	9,686,586	9,711,796	+
SVSP-23	mi-2	10,027,581	10,069,646	+	9,448,868	9,490,933	+	9,724,911	9,766,976	+
SVSP-24	mi-2	10,107,929	10,116,754	+	9,529,216	9,538,041	+	9,805,259	9,814,084	+
PLA2-B	mi-8	10,620,682	10,622,277	–	9,790,718	9,792,313	–	12,156,568	12,158,163	–
PLA2-C	mi-8	10,625,528	10,626,898	–	9,795,564	9,796,934	–	12,161,413	12,162,783	–
PLA2-A	mi-8	10,630,424	10,632,003	+	9,800,460	9,802,039	+	12,166,309	12,167,888	+

Abbreviations: Chr—Chromosome, NA—Not annotated in the indicated assembly, Str—Strand.

Table S2. Comparison of toxin classes annotated in Hogan *et al.* (2024) and considered toxin classes* in the current assemblies.

Toxin family	Paralog locus	Chr	Primary	Hap1	Hap2	Hogan *et al.*
SVMP	SVMP-23-mdc	mi-1	★	★	★	★
	SVMP-22-mdc	mi-1	★	★	★	★
	SVMP-21-mad	mi-1	★	★	★	★
	SVMP-20-mpo	mi-1	★	★	★	★
	SVMP-19-mdc	mi-1	★	★	★	★
	SVMP-18-mdc	mi-1	★	★	★	★
	SVMP-17-mdc	mi-1	★	★	★	★
	SVMP-16-mdc	mi-1	★	–	★	★
	SVMP-15-mad	mi-1	★	–	★	★
	SVMP-14-mad	mi-1	★	–	★	★
	SVMP-13-mdc	mi-1	★	–	★	★
	SVMP-12-mdc	mi-1	★	–	★	★
	SVMP-11-mdc	mi-1	★	–	★	★
	SVMP-10-mdc	mi-1	★	★	★	★
	SVMP-9-mad	mi-1	★	★	★	★
	SVMP-8-mad	mi-1	★	★	★	★
	SVMP-7-mdc	mi-1	★	★	★	★
	SVMP-6-mad	mi-1	★	★	★	★
	SVMP-5-mad	mi-1	★	★	★	★
	SVMP-4-mad	mi-1	★	★	★	★
	SVMP-3-mdc	mi-1	★	★	★	★
	SVMP-2-mdc	mi-1	★	★	★	★
	SVMP-1-mdc	mi-1	★	★	★	★
SVSP	SVSP-1	mi-2	★	★	★	★
	SVSP-2	mi-2	★	★	–	–
	SVSP-3	mi-2	★	–	–	–
	SVSP-4	mi-2	★	–	–	–
	SVSP-5	mi-2	★	–	–	–
	SVSP-6	mi-2	★	–	–	–
	SVSP-7	mi-2	★	–	★	–
	SVSP-8	mi-2	★	–	★	–
	SVSP-9	mi-2	★	–	★	–
	SVSP-10	mi-2	★	–	★	–
	SVSP-11	mi-2	★	★	★	★
	SVSP-12	mi-2	★	★	★	★
	SVSP-13	mi-2	★	★	★	★
	SVSP-14	mi-2	★	★	★	★
	SVSP-15	mi-2	★	★	★	★
	SVSP-16	mi-2	★	★	★	★
	SVSP-17	mi-2	★	★	★	★
	SVSP-18	mi-2	★	★	★	★
	SVSP-19	mi-2	★	★	★	★
	SVSP-20	mi-2	★	★	★	★
	SVSP-21	mi-2	★	★	★	★
	SVSP-22	mi-2	★	★	★	★
	SVSP-23	mi-2	★	★	★	★
	SVSP-24	mi-2	★	★	★	★
PLA2	PLA2-B	mi-8	★	★	★	∃
	PLA2-C	mi-8	★	★	★	★
	PLA2-A	mi-8	★	★	★	★
CTL	CTL-1-beta	ma-6	★	★	★	★
	CTL-2-alpha	ma-6	★	★	★	★
	CTL-3-beta	ma-6	★	★	★	★
	CTL-4-alpha	ma-6	★	★	★	★
	CTL-5-beta	ma-6	★	★	★	★
	CTL-6-alpha	ma-6	★	★	★	★
	CTL-7-beta	ma-6	★	★	★	★
	CTL-8-alpha	ma-6	★	★	★	★
	CTL-9-beta	ma-6	★	★	★	★
	CTL-10-lectin	ma-1	★	★	★	★
MYO	MYO-1	ma-2	★	★	★	★
	MYO-2	ma-2	★	★	★	★
	MYO-3	ma-2	★	★	★	★
	MYO-4	ma-2	★	★	★	★
	VENOM-MYO-2	ma-2	–	–	–	★
KUN	KUN-1	ma-1	★	★	★	★
	KUN-2	mi-2	★	★	★	★
CRISP	CRISP-1	ma-1	★	★	★	★
	CRISP-2	ma-1	∃	∃	∃	★
	CRISP-3	ma-1	∃	∃	∃	★
	CRISP-4	ma-1	∃	∃	∃	★

NUC	NUC-1	ma-1	★	★	★	★
PDE	PDE-1	ma-1	★	★	★	★
	PDE-2	ma-1	∃	∃	∃	★
VEGF	VEGF-A	ma-1	★	★	★	★
Vespryn	Vespryn-1	ma-2	★	★	★	★
LAAO	LAAO-1	ma-2	★	★	★	★
	LAAO-2	ma-2	★	★	★	★
NGF	NGF-1	ma-3	★	★	★	★
NP	BPP-1	ma-5	★	★	★	★
HYAL	HYAL-1	ma-6	★	★	★	★
PLB	PLB-1	ma-6	★	★	★	★

*We considered the review performed by Oliveira *et al.* (2022), venom-gland expression levels, and presence in the venom proteome to determine whether to consider a paralog as a toxin.
★ ⇒ detected and annotated as a toxin in that assembly.
∃ ⇒ detected and not considered as a toxin in that assembly.
– ⇒ not detected in that assembly.
Abbreviations: Chr—Chromosome, ma—Macrochromosome, mi—Microchromosome.

Table S3. Comparison of toxin classes annotated in Hogan *et al.* (2024) and not considered as toxins classes* in the current assemblies.

Toxin family	Paralog locus	Chr	Primary	Hap1	Hap2	Hogan *et al.*
β-defensin/DEF	β-defensin-01	ma-2	∃	∃	∃	★
	β-defensin-02	ma-2	∃	∃	∃	★
	VENOM-β-defensin-03	ma-2	∃	∃	∃	★
	β-defensin-04	ma-2	∃	∃	∃	★
	β-defensin-05	ma-2	∃	∃	∃	★
	β-defensin-06	ma-2	∃	∃	∃	★
	β-defensin-07	ma-2	∃	∃	∃	★
	β-defensin-08	ma-2	∃	∃	∃	★
	β-defensin-09	ma-2	∃	∃	∃	★
	β-defensin-10	ma-2	∃	∃	∃	★
	β-defensin-11	ma-2	∃	∃	∃	★
	β-defensin-12	ma-2	∃	∃	∃	★
	β-defensin-13	ma-2	∃	∃	∃	★
	β-defensin-14	ma-2	∃	∃	∃	★
	β-defensin-15	ma-2	∃	∃	∃	★
	β-defensin-16	ma-2	∃	∃	∃	★
3FTx	VENOM-3FTx-01	ma-3	∃	∃	∃	★
	VENOM-3FTx-02	ma-3	∃	∃	∃	★
	VENOM-3FTx-03	ma-3	∃	∃	∃	★
	VENOM-3FTx-04	ma-3	∃	∃	∃	★
	3FTx-05	ma-3	∃	∃	∃	★
	3FTx-06	ma-3	∃	∃	∃	★
	VENOM-3FTx-07	ma-3	∃	∃	∃	★
	VENOM-3FTx-08	ma-3	∃	∃	∃	★
	3FTx-09	ma-3	∃	∃	∃	★
	3FTx-10	ma-3	∃	∃	∃	★
	3FTx-11	ma-3	∃	∃	∃	★
	3FTx-12	ma-3	∃	∃	∃	★
	3FTx-13	ma-3	∃	∃	∃	★
	3FTx-14	ma-3	∃	∃	∃	★
	3FTx-15	ma-3	∃	∃	∃	★
	VENOM-3FTx-16	ma-3	∃	∃	∃	★
	VENOM-3FTx-17	ma-3	∃	∃	∃	★
	3FTx-18	ma-3	∃	∃	∃	★
	VENOM-3FTx-19	ma-3	∃	∃	∃	★
	3FTx-20	ma-3	∃	∃	∃	★
	3FTx-21	ma-3	∃	∃	∃	★
	3FTx-22	ma-3	∃	∃	∃	★
	3FTx-23	ma-3	∃	∃	∃	★
	3FTx-24	ma-3	∃	∃	∃	★
	3FTx-25	ma-3	∃	∃	∃	★
	3FTx-26	ma-3	∃	∃	∃	★
	3FTx-27	ma-3	∃	∃	∃	★
	3FTx-28	ma-3	∃	∃	∃	★
	3FTx-29	ma-3	∃	∃	∃	★
	3FTx-30	ma-3	∃	∃	∃	★
	3FTx-31	ma-3	∃	∃	∃	★
	3FTx-32	ma-3	∃	∃	∃	★
	3FTx-33	ma-3	∃	∃	∃	★

*We considered the review performed by Oliveira *et al.* (2022), venom-gland expression levels, and presence in the venom proteome to determine whether to consider a paralog as a toxin.
★ ⇒ detected and annotated as a toxin in that assembly.
∃ ⇒ detected and not considered as a toxin in that assembly.
– ⇒ not detected in that assembly.
Abbreviations: Chr—Chromosome, ma—Macrochromosome, mi—Microchromosome.

Table S4. Summary of genomic and transcriptomic datasets from *C. adamanteus* used in the present study.

Type	Sample	Sex	Tissue	FL county	Accession	Objective	Reference
WGS-HiFi	**DRR0105**	Female	Blood	Leon	SRR21092035, SRR21092036	Genome assembly	Hogan *et al.* (2024)
HiC	**DRR0105**	Female	Blood	Leon	SRR28357486	Genome assembly	present study
WGS-SR	KW0944	Male	Blood	Miami-Dade	SRR12802469	Confirm sex chromosomes	Hogan *et al.* (2021)
WGS-SR	KW1264	Female	Blood	Leon	SRR12802470	Confirm sex chromosomes	Hogan *et al.* (2021)
RNA-seq	KW0944	Male	Gonad	Miami-Dade	SRR21096550	Genome annotation	Hogan *et al.* (2021)
RNA-seq	KW0944	Male	Heart	Miami-Dade	SRR21096549	Genome annotation	Hogan *et al.* (2021)
RNA-seq	KW0944	Male	Kidney	Miami-Dade	SRR21096546	Genome annotation	Hogan *et al.* (2021)
RNA-seq	KW0944	Male	Venom gland	Miami-Dade	SRR21096543	Genome annotation	Hogan *et al.* (2021)
RNA-seq	KW0944	Male	Liver	Miami-Dade	SRR21096545	Genome annotation	Hogan *et al.* (2021)
RNA-seq	KW0944	Male	Muscle	Miami-Dade	SRR21096544	Genome annotation	Hogan *et al.* (2021)
RNA-seq	KW1264	Female	Gonad	Leon	SRR21096542	Genome annotation	Hogan *et al.* (2021)
RNA-seq	KW1264	Female	Heart	Leon	SRR21096541	Genome annotation	Hogan *et al.* (2021)
RNA-seq	KW1264	Female	Kidney	Leon	SRR21096540	Genome annotation	Hogan *et al.* (2021)
RNA-seq	KW1264	Female	Venom gland	Leon	SRR21096547	Genome annotation	Hogan *et al.* (2021)
RNA-seq	KW1264	Female	Liver	Leon	SRR21096539	Genome annotation	Hogan *et al.* (2021)
RNA-seq	KW1264	Female	Muscle	Leon	SRR21096548	Genome annotation	Hogan *et al.* (2021)
RNA-seq	**DRR0105**	Female	Venom gland	Leon	SRR12915694	Estimate toxin expression	Hogan *et al.* (2024)
RNA-seq	DRR0044	Male	Venom gland	Leon	SRR21096589	Estimate toxin expression	Hogan *et al.* (2024)
RNA-seq	DRR0106	Male	Venom gland	Leon	SRR12915692	Estimate toxin expression	Hogan *et al.* (2024)
RNA-seq	DRR0107	Male	Venom gland	Liberty	SRR21096588	Estimate toxin expression	Hogan *et al.* (2024)
RNA-seq	DRR0108	Female	Venom gland	Leon	SRR21096587	Estimate toxin expression	Hogan *et al.* (2024)
RNA-seq	KW0944	Male	Venom gland	Miami-Dade	SRR5259500, SRR5259499	Estimate toxin expression	Hogan *et al.* (2024)
RNA-seq	KW1264	Female	Venom gland	Leon	SRR5259498, SRR5259497	Estimate toxin expression	Hogan *et al.* (2024)
RNA-seq	KW1942	Male	Venom gland	Pinellas	SRR5259496, SRR5259495	Estimate toxin expression	Hogan *et al.* (2024)
RNA-seq	KW2161	Male	Venom gland	Franklin	SRR5259494, SRR5259493	Estimate toxin expression	Hogan *et al.* (2024)
RNA-seq	KW2170	Female	Venom gland	Pinellas	SRR5259492, SRR5259491	Estimate toxin expression	Hogan *et al.* (2024)
RNA-seq	KW2171	Male	Venom gland	Hernando	SRR5259490, SRR5259489	Estimate toxin expression	Hogan *et al.* (2024)
RNA-seq	KW2184	Female	Venom gland	Franklin	SRR5259488, SRR5259487	Estimate toxin expression	Hogan *et al.* (2024)
RNA-seq	MM0114	Female	Venom gland	Leon	SRR12915690	Estimate toxin expression	Hogan *et al.* (2024)
RNA-seq	MM0127	Male	Venom gland	Hernando	SRR5259486, SRR5259485	Estimate toxin expression	Hogan *et al.* (2024)
RNA-seq	MM0143	Male	Venom gland	Miami-Dade	SRR5259484, SRR5259483	Estimate toxin expression	Hogan *et al.* (2024)
RNA-seq	MM0198	Female	Venom gland	Leon	SRR5259482, SRR5259481	Estimate toxin expression	Hogan *et al.* (2024)
RNA-seq	TJC1661	Male	Venom gland	Liberty	SRR21096586	Estimate toxin expression	Hogan *et al.* (2024)
RNA-seq	TJC1665	Male	Venom gland	Liberty	SRR21096585	Estimate toxin expression	Hogan *et al.* (2024)

Samples of the individual used to assemble the genome are highlighted in bold. The “FL county” column provides the Florida county where the specimen was sampled. Abbreviations: WGS-HiFi—whole genome sequencing using PacBio Hifi method, WGS-SR—whole genome sequencing using short-reads method, HiC—Hi-C method, RNA-seq—transcriptomic data.

References

Giorgianni MW, Dowell NL, Griffin S, Kassner VA, Selegue JE, Carroll SB. The origin and diversification of a novel protein family in venomous snakes. *Proc Natl Acad Sci USA*. 2020:**117**(20):10911–10920. https://doi.org/10.1073/pnas.1920011117.

Hogan MP, Holding ML, Nystrom GS, Colston TJ, Bartlett DA, Mason AJ, Ellsworth SA, Rautsaw RM, Lawrence KC, Strickland JL, *et al.* The genetic regulatory architecture and epigenomic basis for age-related changes in rattlesnake venom. *Proc Natl Acad Sci USA*. 2024:**121**(16):e2313440121. https://doi.org/10.1073/pnas.2313440121.

Hogan MP, Whittington AC, Broe MB, Ward MJ, Gibbs HL, Rokyta DR. The chemosensory repertoire of the eastern diamondback rattlesnake (*Crotalus adamanteus*) reveals complementary genetics of olfactory and vomeronasal-type receptors. *J Mol Evol*. 2021:**89**(6):313–328. https://doi.org/10.1007/s00239-021-10007-3.

Margres MJ, Bigelow AT, Lemmon EM, Lemmon AR, Rokyta DR. Selection to increase expression, not sequence diversity, precedes gene family origin and expansion in rattlesnake venom. *Genetics*. 2017:**206**(3):1569–1580. https://doi.org/10.1534/genetics.117.202655.

Margres MJ, Patton A, Wray KP, Hassinger AT, Ward MJ, Lemmon EM, Lemmon AR, Rokyta DR. Tipping the scales: The migration–selection balance leans toward selection in snake venoms. *Mol Biol Evol*. 2019:**36**(2):271–282. https://doi.org/10.1093/molbev/msy207.

Margres MJ, Rautsaw RM, Strickland JL, Mason AJ, Schramer TD, Hofmann EP, Stiers E, Ellsworth SA, Nystrom GS, Hogan MP, *et al.* The Tiger Rattlesnake genome reveals a complex genotype underlying a simple venom phenotype. *Proc Natl Acad Sci USA*. 2021:**118**(4):e2014634118. https://doi.org/10.1073/pnas.2014634118.

Oliveira AL, Viegas MF, da Silva SL, Soares AM, Ramos MJ, Fernandes PA. The chemistry of snake venom and its medicinal potential. *Nat Rev Chem*. 2022:**6**(7):451–469. https://doi.org/10.1038/s41570-022-00393-7.

Vollger MR, Dishuck PC, Sorensen M, Welch AE, Dang V, Dougherty ML, Graves-Lindsay TA, Wilson RK, Chaisson MJ, Eichler EE. Long-read sequence and assembly of segmental duplications. *Nat Methods*. 2019:**16**(12):88–94. https://doi.org/10.1038/s41592-018-0236-3.
